# Better prediction of clinical outcome in clear cell renal cell carcinoma based on a 6 metabolism-related gene signature

**DOI:** 10.1038/s41598-023-38380-7

**Published:** 2023-07-17

**Authors:** Zhixian Yu, Yating Zhan, Yong Guo, Dalin He

**Affiliations:** 1 Department of Urology, The First Affiliated Hospital of Xi’an Jiaotong University, 277 Yanta West Road, Xi’an, 710061 China; 2 Department of Urology, The First Affiliated Hospital of Wenzhou Medical University, Wenzhou, China; 3 Key Laboratory of Diagnosis and Treatment of Severe Hepato-Pancreatic Diseases of Zhejiang Province, The First Affiliated Hospital of Wenzhou Medical University, Wenzhou, China

**Keywords:** Cancer, Computational biology and bioinformatics, Nephrology

## Abstract

It has been reported that metabolic disorders participate in the formation and progression of clear cell renal cell carcinoma (ccRCC). However, the predictive value of metabolism-related genes (MRGs) in clinical outcome of ccRCC is still largely unknown. Herein, a novel metabolism-related signature was generated to assess the effect of MRGs on the prognosis of ccRCC patients. Important module MRGs were selected by differentially expressed analysis and WGCNA. Subsequently, the hub MRGs were screened via univariate cox regression as well as LASSO regression. A new metabolism-related signature of 6 hub MRGs (PAFAH2, ACADSB, ACADM, HADH, PYCR1 and ITPKA) was constructed, with a good prognostic prediction ability in the TCGA cohort. The prediction accuracy of this signature was further confirmed in both GSE22541 and FAHWMU cohort. Interestingly, this MRG risk signature was highly correlated with tumor mutation burden and immune infiltration in ccRCC. Notably, lower PAFAH2, a member of 6 MRGs, was found in ccRCC. Knockdown of PAFAH2 contributed to renal cancer cell proliferation and migration. Collectively, a 6-MRG prognostic risk signature is generated to estimate the prognostic status of ccRCC patients, providing a novel insight in the prognosis prediction and treatment of ccRCC.

## Introduction

Renal cell carcinoma (RCC) is known as a common human renal malignancy, especially in adults^1^. In comparison with other RCC subtypes, clear cell renal cell carcinoma (ccRCC) has a worse survival outcome^2,3^. Radical nephrectomy is an important intervention for localized RCC, while systemic therapy is the main therapy for patients with advanced RCC^4^. Due to RCC resistance in radiotherapy and chemotherapy, immunotherapy has been developed for treating RCC patients. The use of immune checkpoint inhibitors (ICIs) alone or combination has exhibited good therapeutic effects on ccRCC^5^. However, ccRCC patients generally exhibit different therapeutic effects due to individual differences. As a result, an effective signature is needed to generate to evaluate the prognosis of ccRCC patients with effective ICIs or drugs.

Metabolism disorder has been found in multiple tumors^6^. Imbalance of various metabolites has been reported to be highly related with ccRCC tumorigenesis and progression. For instance, it has been found that disorder of glucose/fatty acid metabolism as well as tricarboxylic acid cycle often contributes to the initiation of ccRCC^7^. Hiromi et al. revealed that RCC cells mainly depend on aerobic glycolysis (Warburg effect), and glycolysis-relevant metabolites are increased with higher grade^8^. Moreover, enhanced cholesterol ester storage is found in patients with ccRCC^9^. Therefore, an in-depth study of metabolism-related signature may contribute to comprehending the potential roles of metabolism-related genes (MRGs) in the prediction of ccRCC prognosis.

Herein, a 6-MRG prognostic risk signature was constructed to evaluate ccRCC prognosis. Patients with low-risk were shown to have a better overall survival (OS). The accuracy and specificity of our signature was further validated in the GSE22541 cohort and the First Affiliated Hospital of Wenzhou Medical University (FAHWMU) cohort. The immune status of ccRCC patients with different risk score was further explored. Furthermore, the targeted treatment of potential chemotherapeutic agents and ICIs therapies in ccRCC was investigated. Several chemotherapeutic agents and ICIs were shown to have a good performance in the high-risk group. Therefore, our study demonstrates the potential prognostic value of the 6-MRG prognostic risk signature, which may provide a novel insight in ccRCC treatment and be promising biomarkers for ccRCC progression.

## Results

### Identification of survival-related differentially expressed MRGs (DEMRGs)

**Figure 1:**
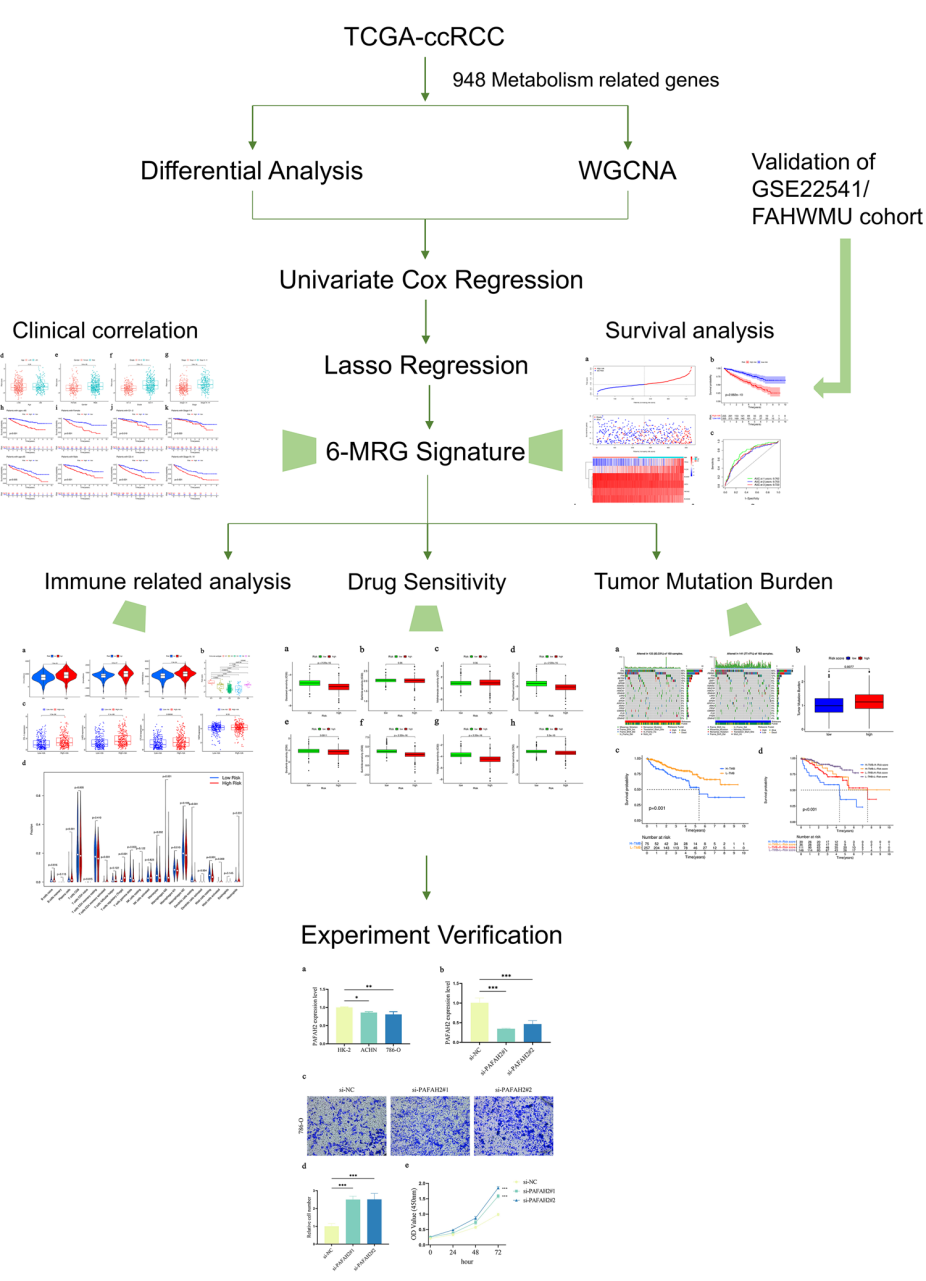
Flow diagram of the data analysis procedure.

**Figure 2:**
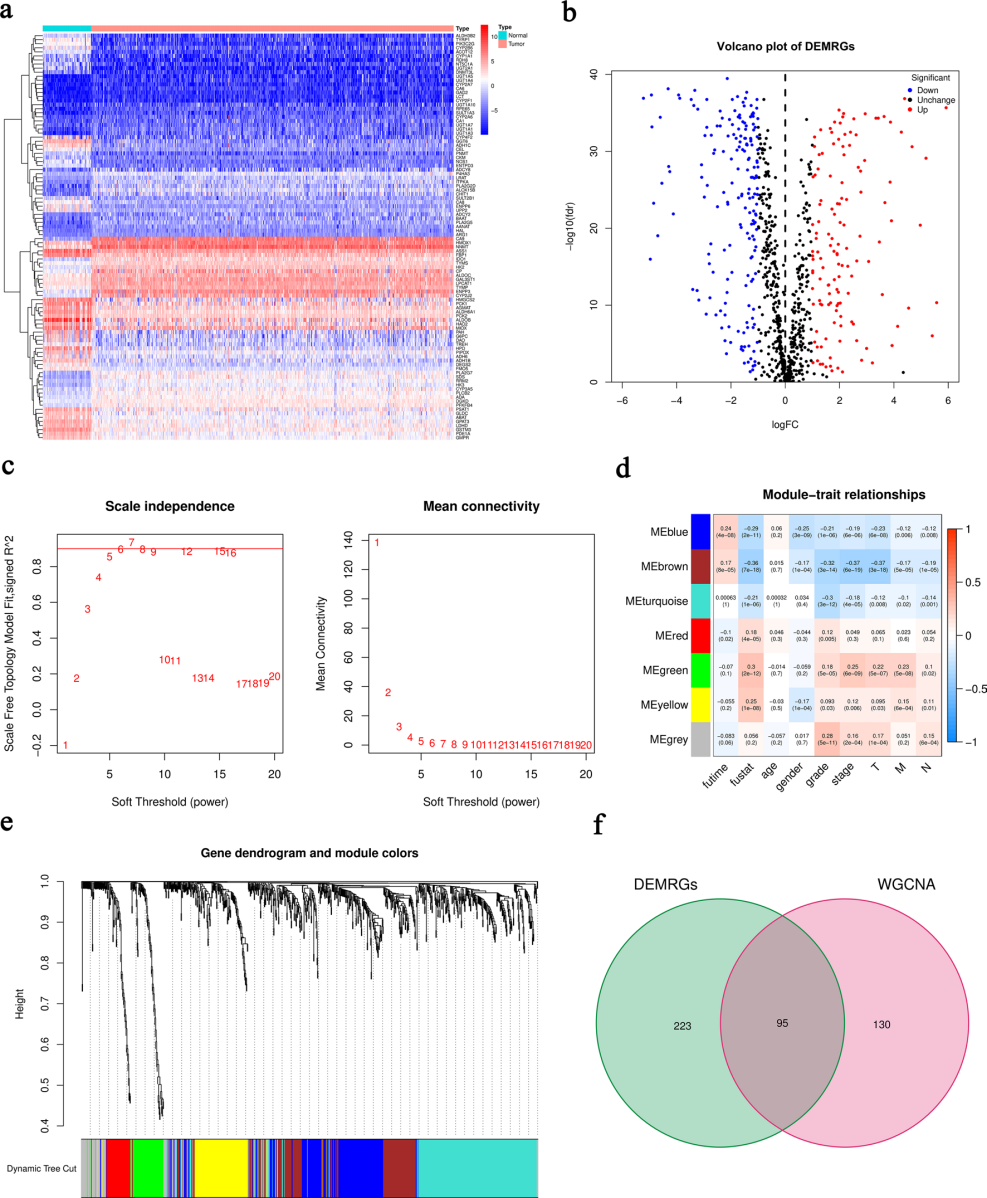
Identification of survival-related DEMRGs. (**a**) Heatmap of top 100 DEMRGs. (**b**) Volcano plot of DEMRGs. (**c**) Network topology with different soft threshold powers. (**d**) Heatmap of the correlation between module genes and clinical factors. (**e**) Cluster diagram. (**f**) The intersection between DEMRGs and key module MRGs.

The overall work flow was shown in Fig. 1. To explore the role of metabolism pathway in ccRCC, a total of 948 MRGs were obtained from the Kyoto Encyclopedia of Genes and Genomes (KEGG) pathway related with kinds of substance metabolism. Then, the mRNA expressions of 948 MRGs were extracted from the Cancer Genome Atlas (TCGA) and 318 DEMRGs were obtained. The corresponding heatmap and volcano plot were shown in Fig. 2a,b. The detailed information of 318 DEMRGs were listed in Table S1. Then, weighted gene co-expression network analysis (WGCNA) was performed to identify key survival-related modules in 948 MRGs. The soft threshold power was considered as 5 according to scale-free topology R^2^ and mean connectivity (Fig. 2c). Then, the clustering dendrograms were used to display the results of the combined 7 modules (Fig. 2e). The relationships between modules and clinical factors demonstrated that there was a positive correlation between green module and survival status, and a negative correlation between brown module and survival status (Fig. 2d). Additionally, correlations between other clinical factors and green as well as brown module were similar with the above results. Next, 225 genes, extracted from green and brown modules, were used to be intersected with DEMRGs. Finally, 95 survival-related DEMRGs were selected (Fig. 2f).

### Selection of prognostic MRGs

To identify the prognostic value of MRGs in ccRCC, we included 95 survival-related DEMRGs into univariate cox regression analysis. Then, 54 prognostic MRGs (including 21 dangerous MRGs and 33 protective MRGs) were obtained according to *p* < 0.05 (Table S2). To further investigate the underlying functions and pathways of the 54 prognostic MRGs, Gene Ontology (GO) and KEGG analyses were performed. The results of GO indicated that prognostic MRGs were mainly enriched in molecule-related catabolic processes (Fig. S1a). KEGG analysis disclosed that prognostic MRGs were mainly associated with metabolism-related pathways in ccRCC (Fig. S1b). The results of enrichment analysis indicated that prognostic MRGs may influence the progression of ccRCC via the above metabolic pathways. Subsequently, we analyzed the relationships between MRGs and tumor-related transcription factors (TFs) to uncover the potential regulatory mechanisms of prognostic MRGs. Then, 318 tumor-related TFs from Cistrome were performed with differentially expressed analysis and 60 differentially expressed TFs (DETFs) were screened (Table S3). Thus, we constructed a TF-MRG regulatory network to explore their underlying mechanisms (Fig. S1c).

### Establishment and validation of 6-MRG prognostic risk signature

**Figure 3:**
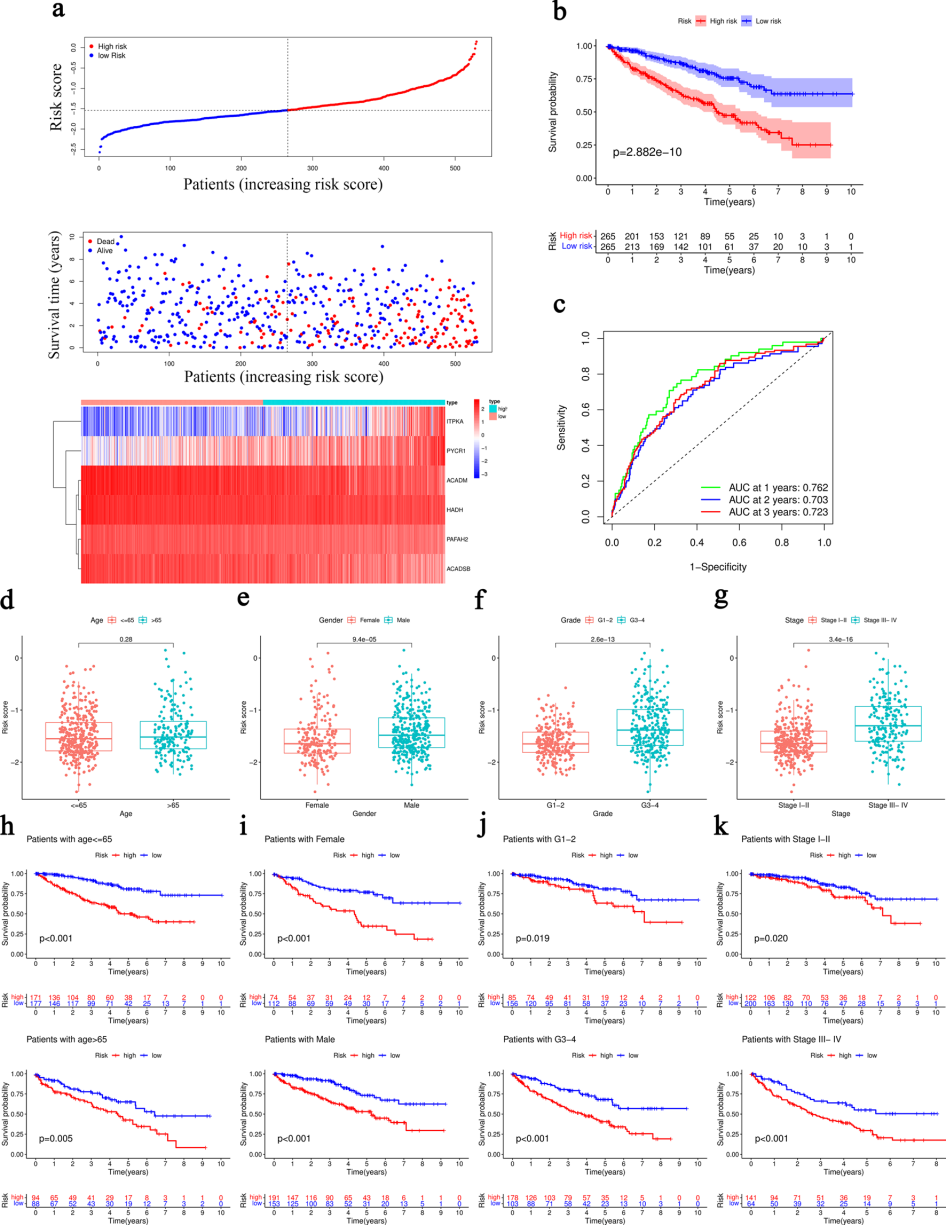
Construction of the 6-MRG prognostic risk signature with OS of ccRCC. (**a**) Distribution of risk score (upper), survival status (middle) and 6-MRG expression profiles (bottom) for all patients in the TCGA cohort. (**b**) Kaplan–Meier curve of OS in the TCGA cohort. (**c**) ROC curve for the TCGA cohort. (**d**–**g**) Box plots of the correlation between risk score and different clinical features (age, gender, grade and stage). (**h**–**k**) Survival analysis.

In order to construct a robust MRG prognostic risk signature without overfitting genes, least absolute shrinkage and selection operator (LASSO) regression analysis was performed with the above 54 prognostic MRGs (Fig. S2). 6 hub MRGs with corresponding LASSO coefficients were selected out for the establishment of risk score formula: Risk score = (− 0.1652 * PAFAH2) + (− 0.0669 * ACADSB) + (− 0.0694 * ACADM) + (− 0.2394 * HADH) + (0.1851 * PYCR1) + (0.1504 * ITPKA). ccRCC patients in TCGA were divided into the high- and low-risk groups according to the median risk score as the cut-off value. The distribution of risk score and survival status as well as expression heatmap of 6 MRGs was shown in Fig. 3a. The results of Kaplan–Meier curve indicated that ccRCC patients in the low-risk group showed a better OS in comparison with patients with high-risk (Fig. 3b). Moreover, the receiver operating characteristic (ROC) curve was employed to assess the accuracy of the 6-MRG risk signature (Fig. 3c). The area under the ROC curve (AUC) value was 0.762 at 1-year, 0.703 at 2-year and 0.723 at 3-year, respectively, suggesting a good sensitivity and specificity of this risk signature. We further validated the accuracy of 6-MRG risk signature in the GSE22541 and FAHWMU cohorts. Consistent with the results of TCGA, the OS of patients with low-risk was better, with 1-year AUC value was 0.672 in the GSE22541 cohort and 0.650 in the FAHWMU cohort, respectively (Fig. S3).

Interestingly, the high-risk group had a significantly higher percentage of ccRCC patients with worse clinicopathological characteristics, such as an advanced tumor stage and a later histological grade (Fig. 3d,e,f,g). Kaplan–Meier curve analysis demonstrated that the prognosis of patients with high-risk was worse than those with low-risk in all the clinical subgroups (Fig. 3h,i,j,k). In addition, the mRNA expressions of 6 MRGs were associated with OS of ccRCC patients (Fig. S4). Overall, the above results demonstrate that our signature may be useful for predicting prognosis in patients with ccRCC.

### Functional enrichment analysis of 6-MRG prognostic risk signature

**Figure 4:**
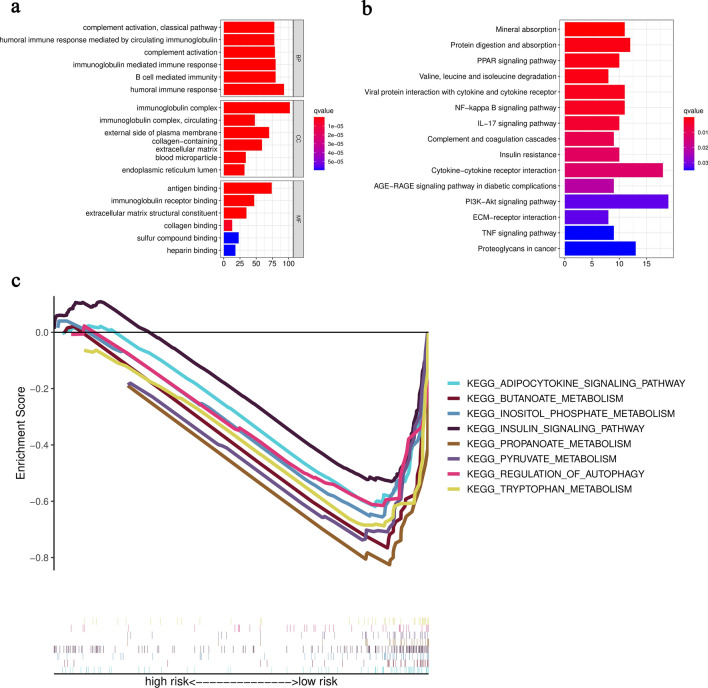
Functional enrichment analysis of 6-MRG prognostic risk signature. (**a**) GO analysis. (**b**) KEGG analysis. (**c**) GSEA analysis.

628 differentially expressed risk genes (DERGs) were obtained via differentially expressed analysis between the high- and low-risk groups. The enrichment analysis of GO and KEGG was conducted to identify the potential biological function and signaling pathways of DERGs. Results of GO indicated that DERGs were significantly enriched in immunoglobulin-mediated immune response, B cell-mediated immunity and humoral immune response, which were strongly associated with immune system (Fig. 4a). Results of KEGG demonstrated the involvement of DERGs in NF-κB signaling pathway, PPAR signaling pathway and PI3K-Akt signaling pathway (Fig. 4b). The results of Gene Set Enrichment Analysis (GSEA) indicated the enrichment of metabolism-related pathways such as adipocytokine signaling pathway, butanoate metabolism, inositol phosphate metabolism, insulin signaling pathway, propanoate metabolism, pyruvate metabolism, regulation of autophagy and tryptophan metabolism in the low-risk group (Fig. 4c). Overall, our risk signature may be associated with immune processes and metabolism pathways in ccRCC.

### Construction of a nomogram signature

The results of univariate cox regression showed that clinical features (age, grade, stage, T, M, and N) and risk score were related with OS (Fig. S5a). Moreover, age and risk score were considered as the independent prognostic factors in ccRCC via multivariate cox regression analysis (Fig. S5b). Then, a nomogram including age and risk score was established to launch more accurate personalized prediction in ccRCC prognosis (Fig. S5c). The results of calibration curve demonstrated that the predictive outcome of nomogram was consistent with the actual outcome (Fig. S5d).

### Immune-related analysis in 6-MRG prognostic risk signature

**Figure 5:**
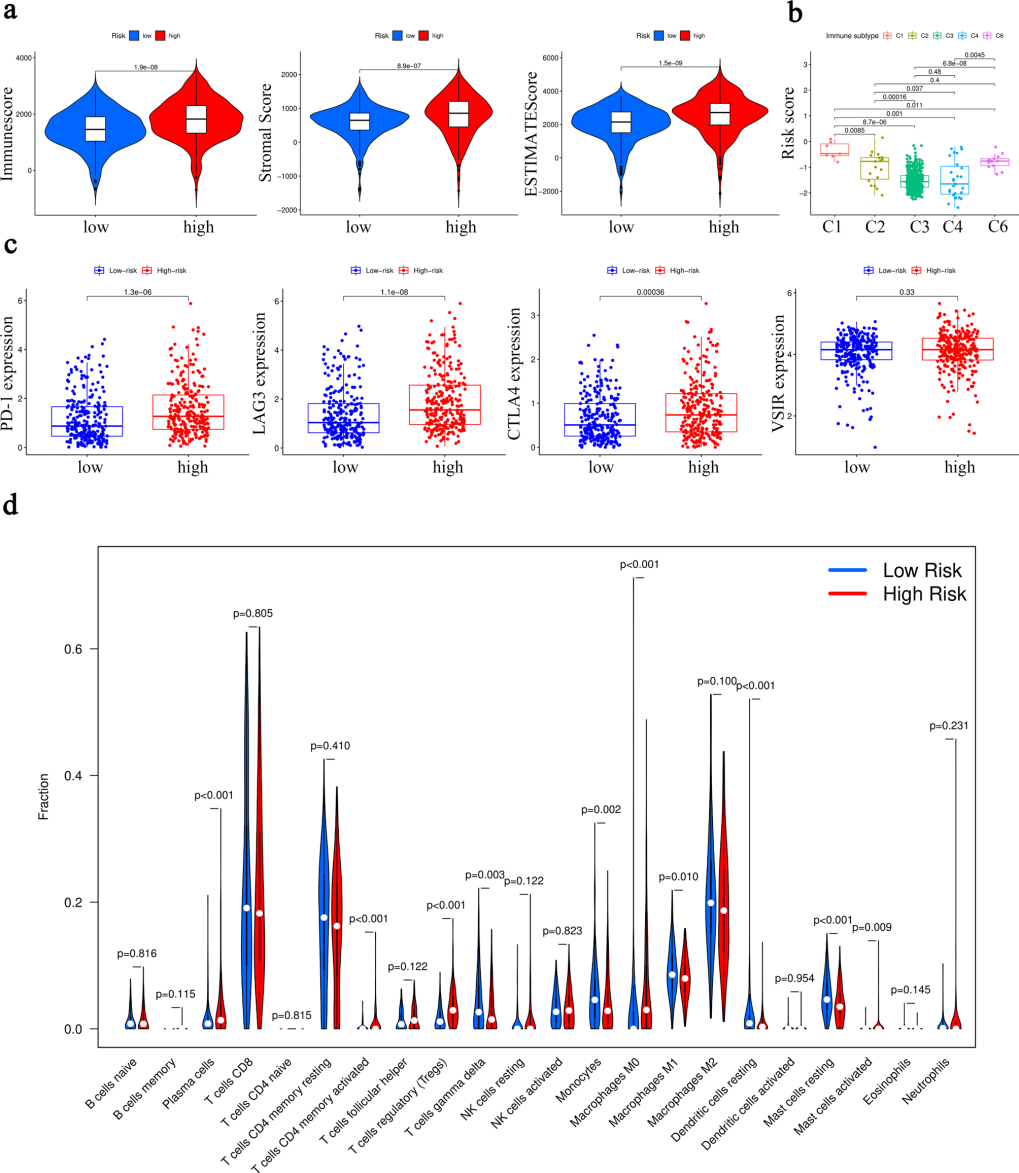
Association between risk score and immune characteristics. (**a**) Distribution of TME scores. (**b**) Relation between immune subtypes and risk score. (**c**) Expression levels of 4 immune checkpoints (PD-1, LAG3, CTLA4 and VSIR). (**d**) Infiltration levels of 22 immune cells.

It has been reported that tumor microenvironment (TME) is involved in the progression of ccRCC^10^. Therefore, the relations between risk signature and TME scores (immune score, stromal score as well as ESTIMATE score) were further investigated. Our results showed that increased TME scores were observed in patients with high-risk compared with those with low-risk (Fig. 5a). Then, analysis of relations between immune subtypes and risk score indicated lower risk score in C2 (IFN-gamma Dominant), C3 (Inflammatory), C4 (Lymphocyte Depleted) and C6 (TGF-beta Dominant) in comparison with C1 (Wound Healing) (Fig. 5b). No ccRCC patients were included into C5 immune subtype. Recently, immunotherapy has been performed a good effect in ccRCC patients^11^. It is important to explore levels of immune checkpoints in ccRCC patients. As shown in Fig. 5c, higher levels of PD-1, LAG3 and CTLA4 were found in patients with high-risk, indicating a positive correlation between risk score and immune checkpoints. Our results suggest that immune checkpoints may be potential therapeutic targets of immunotherapy for ccRCC patients. Next, the infiltration levels of immune cells were further investigated. Higher levels of plasma cells, T cells regulatory (Tregs) and macrophages M0 were observed in patients with high-risk. Moreover, patients with low-risk were correlated with monocytes, macrophages M1, resting dendritic cells and resting mast cells (Fig. 5d). The above results reveal that this MRG risk signature may be associated with immune system in the development of ccRCC.

### Correlation between tumor mutation burden (TMB) and 6-MRG prognostic risk signature

**Figure 6:**
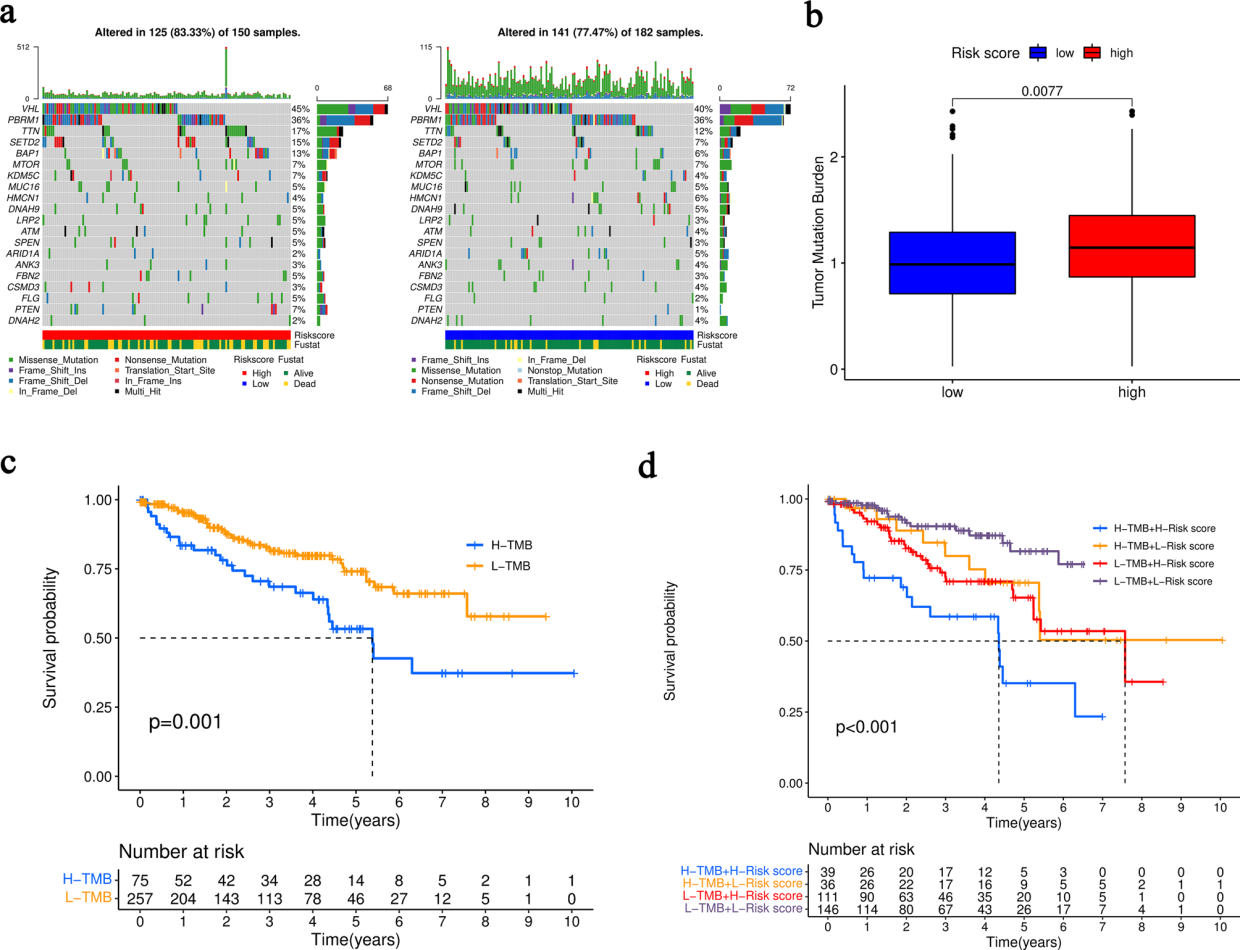
TMB analysis in the TCGA cohort. (**a**) Waterfall plot of mutated genes. (**b**) Difference analysis of TMB. (**c**) Kaplan–Meier curve of OS. (**d**) Kaplan–Meier curve for combined analysis of TMB and risk score.

Gene mutation is considered as a key factor in tumorigenesis and process. Level of TMB in the high- and low-risk groups was analyzed. Waterfall plot displayed the top 20 genes of highest mutation frequency in the high- and low-risk groups (Fig. 6a). Notably, the patients with high-risk had higher gene mutation frequency compared with those with low-risk. Moreover, the level of TMB was remarkably higher in the high-risk group (Fig. 6b). Obviously, the OS of patients with high TMB was worse (Fig. 6c). Furthermore, patients with high-TMB and high-risk score showed the worst OS among all the groups, suggesting that this 6-MRG prognostic risk signature is correlated with TMB in ccRCC progression (Fig. 6d).

### Analysis of drug sensitivity

**Figure 7:**
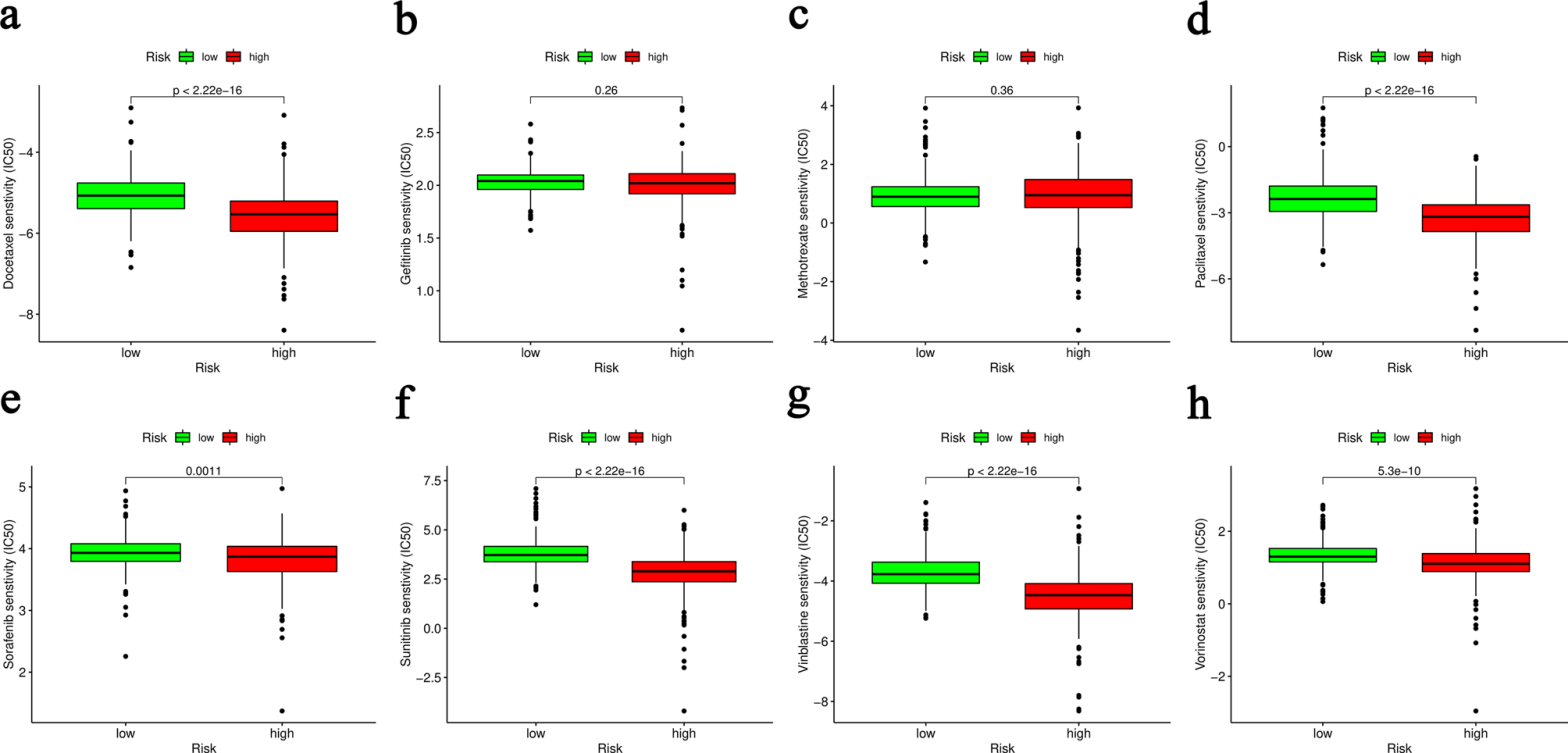
Estimation of chemotherapy response for ccRCC. Docetaxel (**a**), Gefitinib (**b**), Methotrexate (**c**), Paclitaxel (**d**), Sorafenib (**e**), Sunitinib (**f**), Vinblastine (**g**) and Vorinostat (**h**).

To explore the effective chemotherapeutic drugs in ccRCC patients, the analysis of drug sensitivity was applied to predict the potential chemotherapy response of 8 comment drugs between the high- and low-risk groups. It was found that 6 chemotherapeutic drugs (Docetaxel, Paclitaxel, Sorafenib, Sunitinib, Vinblastine and Vorinostat) had a lower inhibitory concentration (IC50) in patients with high-risk (Fig. 7). Our data suggest that Docetaxel, Paclitaxel, Sorafenib, Sunitinib, Vinblastine and Vorinostat may be potential chemotherapeutic drugs for the high-risk group.

### Roles of PAFAH2 in ccRCC

**Figure 8:**
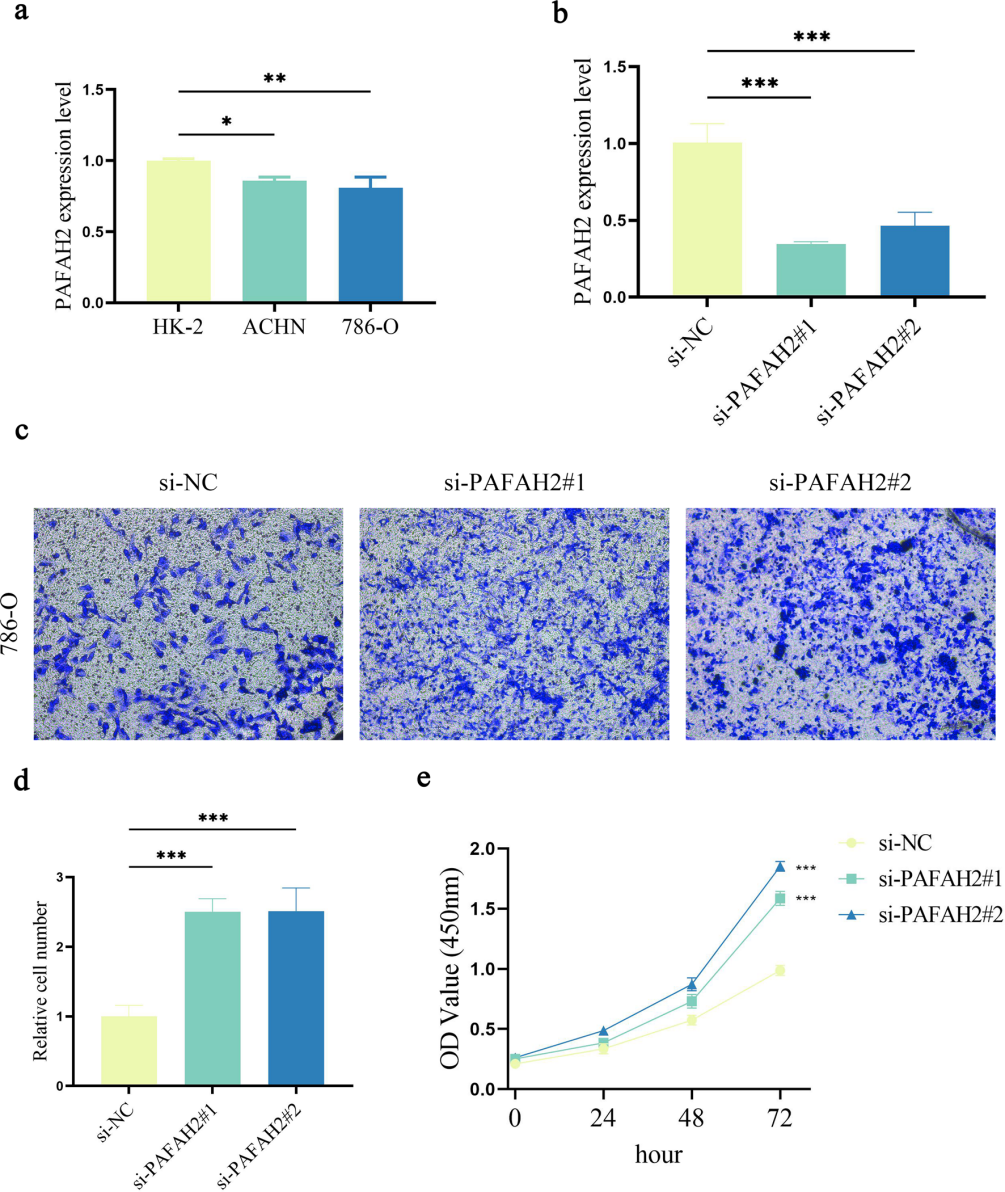
Roles of PAFAH2 in ccRCC. (**a**) Expression level of PAFAH2 in HK-2, ACHN and 786-O cell lines. (**b**) PAFAH2 expression in 786-O cells with transfection of si-NC, si-PAFAH2#1 or si-PAFAH2#2. (**c** and **d)** Transwell assays. (**e**) Cell proliferation. *P < 0.05, **P < 0.01 and ***P < 0.001 compared with the control.

Our 6-MRG prognostic risk signature is composed of PAFAH2, ACADSB, ACADM, HADH, PYCR1 and ITPKA. It is known that the roles of ACADSB, ACADM, HADH, PYCR1 and ITPKA have been explored in cancers, whereas PAFAH2 not^12–16^. Therefore, the mRNA expression and role of PAFAH2 in ccRCC were further investigated. As shown in Fig. 8a, the mRNA expression level of PAFAH2 was reduced in ccRCC cell lines. Clearly, PAFAH2 was inhibited by knockdown of PAFAH2 in 786-O cells (Fig. 8b). Notably, silencing PAFAH2 led to the promotion of cell migration (Fig. 8c,d). Moreover, ccRCC cell proliferation was increased by knockdown of PAFAH2 (Fig. 8e). Combined with these, PAFAH2 may act as a tumor suppressor in ccRCC.

## Discussion

Increasing studies have demonstrated that metabolize dysfunction in glucose, lipid, and amino acid metabolism may contribute to the progression of ccRCC^17^. In addition, dysregulation of MRGs has been reported to be involved in the development of ccRCC. For example, VHL mutation in RCC up-regulates hypoxia-induced vascular endothelial growth factor and promotes nutrition supply in RCC^18^. It has been reported that TP53 mutation can escape RCC cell from the attack of oxidative damage^19^. However, whether MRGs have potential value in the prediction of ccRCC prognosis is still largely unknown. In our study, TF-MRG regulatory network and function enrichment analysis were supplied to systematically understand the potential pathway of prognostic MRGs in ccRCC. Subsequently, a novel 6-MRG prognostic risk signature was generated to contribute to better prediction of the prognosis of ccRCC patients. Our signature indicated novel ccRCC therapeutic targets, providing candidate therapeutic strategies for ccRCC.

This 6-MRG prognostic risk signature contained PAFAH2, ACADSB, ACADM, HADH, PYCR1 and ITPKA. Herein, our data suggest that 6 MRGs may be associated with the prognosis of ccRCC. Lu et al. found that ACADSB, an acyl-CoA dehydrogenase, inhibits the development of colorectal cancer via ferroptosis^20^. ACADM, an enzyme that catalyzes the first step of mitochondrial fatty acid-oxidation pathway, has been shown to inhibit the progression of hepatocellular carcinoma via regulation of CAV1 and SREBP1^21^. Recently, loss of HADH has been found to accelerate cell migration and invasion in gastric cancer^22^. PYCR1, which is often increased in various cancers, has been found to play important roles in regulating cell metabolism including cellular energetic, physiological and pathological processes^23^. PYCR1 has been found to serve as a new prognostic biomarker and therapeutic target for RCC diagnosis and treatment^24^. Previously, Zhu et. al revealed that ITPKA accelerates cell proliferation, migration and invasion of RCC cells via mTORC1^16^. Recently, it has been reported that inhibition of PAFAH2 exacerbates TNF-α-induced lung injury^25^. Due to the reason that the biological roles of PAFAH2 have never been explored in cancers, therefore, the expression and role of PAFAH2 were investigated in ccRCC cells. We demonstrated that in ccRCC cells, reduced PAFAH2 promotes cancer cell proliferation and migration.

Recently, immunotherapy has been developed as an emerging strategy for cancer treatment. Blocking immune checkpoints is an important form of immunotherapy, contributing to activating the antitumor immunity^26^. As a newly developed tumor biomarker, TMB has been a measure of calculating the number of somatic mutations in tumors. Numerous studies have demonstrated that tumors with high TMB exhibit more surface neoantigens and produce more powerful immune response^27,28^. Additionally, high TMB shows a good sensitivity to treatment of ICIs^29^. In this study, the levels of TMB and immune checkpoints (PD-1, LAG3 and CTLA4) were higher in the high-risk group. Together, compared with the low-risk group, patients with high-risk are more likely to benefit from immunotherapy, which provides novel insights in the therapy of ccRCC patients.

It is known that immune infiltration is involved in regulating the development and progression of ccRCC. Recent studies have showed that activated immune cells play key roles in the regulation of immune cells in TME via many metabolic pathways^30–32^. For instance, the inhibition of LOX can reduce the production of CCL2 and IL-10 by tumor-associated macrophages in RCC, leading to the suppression of immune escape and cancer-related inflammation via LOX-dependent arachidonic acid metabolism^33^. Tryptophan catabolism has been demonstrated to produce anti-inflammatory metabolites, contributing to RCC immune suppression^8^. However, the correlation between immune cells and metabolic regulation in ccRCC is still poorly understood. In our study, it was found that levels of plasma cells, M0 macrophage and Tregs were increased in the high-risk group. It is confirmed that immune infiltration is associated with many metabolic pathways.

Docetaxel^34^, Paclitaxel^35^, Sorafenib^36^, Sunitinib^37^, Vinblastine^38^ and Vorinostat^39^ have been demonstrated to have certain effects in RCC therapy. At the present study, patients with high-risk showed higher drug sensitivity with the mentioned 6 drugs. Therefore, our findings suggest that the above drugs may exhibit good effects in the therapy of ccRCC patients with high metabolism score.

Previously, the prognostic value of MRGs has been investigated in various cancers. For instance, Xu et al. proved the prognosis prediction value of a 12-MRG signature in glioma patients^40^. Wu et al. constructed a 11-MRG signature to predict the prognosis of ccRCC patients^41^. Consistent with the previous, our signature was also generated at mRNA expression level. Further studies showed that ITPKA, one member of our 6-MRG risk signature, could not be found in the public-available proteomic data (Clinical Proteomic Tumor Analysis Consortium) (Fig. S6). Therefore, whether our prognostic risk signature applicable at the protein level needs to be explored in the future. In addition, construction of the previous signatures was based on public databases and lack of clinical and experimental verification. There are many advantages in our research. Firstly, a novel 6-MRG prognostic risk signature was established in ccRCC and this is a first report. Secondly, the prognostic value of this signature was validated in the GSE22541 and FAHWMU cohorts. Finally, this signature was correlated with TMB, immune checkpoints and immune infiltration, contributing to the improvement of immunotherapy for ccRCC.

In conclusion, we systematically explore the underlying regulation mechanism of MRGs and their roles in immune-relative pathways of ccRCC. Moreover, we reveal the potential prognostic value of this 6-MRG prognostic risk signature, which may provide novel insights in ccRCC treatment and be promising biomarkers for ccRCC progression.

## Materials and methods

### Data collection

The mRNA data of ccRCC patients were downloaded from TCGA database (https://portal.gdc.cancer.gov/.). In addition, 948 MRGs were extracted from the metabolic pathways of "c2.cp.kegg.v7.4.symbols" in GSEA (https://www.gsea-msigdb.org/gsea/index.jsp.). 530 ccRCC patients in TCGA were used as the training cohort (Table 1). GSE22541 with 24 primary ccRCC patients was supplied from Gene Expression Omnibus (GEO) (https://www.ncbi.nlm.nih.gov/geo/.), as the validation cohort. Moreover, 70 ccRCC patients from FAHWMU were obtained for the accuracy verification of our risk signature. The collection of these ccRCC tissues was agreed by the Ethics Committee of FAHWMU. Informed consents were signed by all the patients/participants in this study.

**Table 1:** Correlations between metabolism signature and clinicopathological parameters of 530 patients in TCGA-ccRCC cohort.

Category	Cases (n = 530)		Metabolism signature	
			Low risk (n = 265)	High risk (n = 265)
Status	Alive	364 (68.68%)	218 (59.89%)	146 (40.11%)
	Death	166 (31.32%)	47 (28.31%)	119 (71.69%)
Age	≤ 65	348 (65.66%)	177 (50.86%)	171 (49.14%)
	> 65	182 (34.34%)	88 (48.35%)	94 (51.65%)
Gender	Female	186 (35.09%)	112 (60.22%)	74 (39.78%)
	Male	344 (64.91%)	153 (44.48%)	191 (55.52%)
Grade	G1	14 (2.64%)	11 (78.57%)	3 (21.43%)
	G2	227 (42.83%)	145 (63.88%)	82 (36.12%)
	G3	206 (38.87%)	95 (46.12%)	111 (53.88%)
	G4	75 (14.15%)	8 (10.67%)	67 (89.33%)
	Unknown	8 (1.51%)		
Stage	Stage I	265 (50.00%)	171 (64.53%)	94 (35.47%)
	Stage II	57 (10.75%)	29 (50.88%)	28 (49.12%)
	Stage III	123 (23.21%)	44 (35.77%)	79 (64.23%)
	Stage IV	82 (15.47%)	20 (24.39%)	62 (75.61%)
	Unknown	3 (0.57%)		
T	T1	271 (51.13%)	173 (63.84%)	98 (36.16%)
	T2	69 (13.02%)	30 (43.48%)	39 (56.52%)
	T3	179 (33.77%)	60 (33.52%)	119 (66.48%)
	T4	11 (2.08%)	2 (18.18%)	9 (81.82%)
M	M0	420 (79.25%)	227 (54.05%)	193 (45.95%)
	M1	78 (14.72%)	19 (24.36%)	59 (75.64%)
	Unknown	32 (6.04%)		
N	N0	239 (45.09%)	121 (50.63%)	118 (49.37%)
	N1	16 (3.02%)	3 (18.75%)	13 (81.25%)
	Unknown	275 (51.89%)		

### Identification of DEMRGs and establishment of weighted gene co-expression networks

The DEMRGs were identified between ccRCC and normal kidney tissues via R package “limma” ((|log_2_ fold change (FC)|> 1, *p* < 0.05))^42^. The key modules with similar expression genes were divided according to WGCNA. In addition, R package "WGCNA" was performed to visualize the modules^43^. Soft threshold power was identified with the scale-free topology R^2^ nearly as 0.90 and the mean connectivity nearly as 0. Gene dendrogram with module colors was displayed after clustering. The module-trait relationship was shown in heatmap to identify clinically relevant MRGs modules. The key survival-related modules were selected based on the associations between modules and clinical features.

### Functional enrichment analysis of prognostic MRGs

The intersection between DEMRGs and key module MRGs was selected. Then, univariate cox regression was performed for the identification of prognostic MRGs, which were significantly correlated with the OS of ccRCC patients. Subsequently, GO and KEGG analyses were employed via R packages “clusterProfiler” and “enrichplot^44^”.

### Construction of the regulatory network between MRGs and TFs

The relationships between MRGs and tumor-related TFs were analyzed to uncover the potential regulatory mechanisms of prognostic MRGs. A total of 318 TFs were obtained through the website Cistrome (http://cistrome.org/). DETFs were filtered via |log_2_FC|> 1 and adjusted *p* < 0.05. Then, the relationships between DETFs and prognostic MRGs were analyzed through correlation test with correlation coefficient > 0.4 and *p* < 0.001. The Cytoscape software was further employed to build a MRG-TF regulatory network.

### Establishment and validation of 6-MRG prognostic risk signature in ccRCC

The TCGA cohort was included to establish the prognostic risk signature. Moreover, the GSE22541 cohort (n = 24) as well as the FAHWMU cohort (n = 70) was used as external verification. Prognostic MRGs were taken into LASSO regression analysis to decrease overfitting genes via R package “glmnet”. The MRG prognostic risk signature was constructed via the LASSO coefficients: $Risk \, score = \sum\nolimits_{i = 1}^{N} {\left( {Exp\left( i \right) \cdot coe\left( i \right)} \right)}$, in which Exp(i) represents the mRNA expression of each gene and coe(i) is the LASSO coefficient of each gene. Based on the risk score formula, the patients were distributed into the low- and high-risk groups using the median risk score as the cut-off value. R packages “survival” and “surviminer” were utilized to analysis the survival status of risk groups. Furthermore, ROC curve and AUC value were also evaluated via R package "timeROC". To confirm the prognostic precision of 6-MRG signature, the risk score in the GSE22541 and FAHWMU cohorts was calculated by risk formula and all patients were assigned into the high- and low-risk groups according to the median risk score from training group. The Kaplan–Meier curve and ROC curve analyses were also performed.

### Functional enrichment analysis of 6-MRG prognostic risk signature

R package “limma” was used to determine DERGs between the high- and low-risk groups in the TCGA cohort (|log_2_FC|> 1, *p* < 0.05). GO and KEGG analyses were performed based on DERGs. R package “clusterProfiler” and “enrichplot” were used for GO and KEGG analyses. GSEA software (GSEA, version 4.0.3) was used for GSEA analysis.

### Independent prognostic analysis

Univariate and multivariate cox regression analyses of clinical characteristics and risk score were performed to determine the independent prognostic factors. R packages “survival” and “survminer” were applied in above analyses via R software. *P* < 0.05 was considered as statistically significant threshold. The independent prognostic factors screened by independent prognostic analysis were subsequently incorporated into the construction of nomogram. Nomogram was established via R package “rms”. We also performed the calibration curve to test the accuracy between the survival probability of nomogram and actual observation value.

### TMB analysis

TMB, based on somatic mutation data in each tumor, is calculated as the total number of abnormal errors per million bases of genes^45^. Mutation data were available in the TCGA database (https://portal.gdc.cancer.gov/). We filtered the mutation types and removed some mutations that could not be reflected on the transcriptome. In addition, only ccRCC patients with both mutation data and prognostic data were included in this study. Perl scripts were used to calculate TMB score for each ccRCC patient. All ccRCC patients were classified into two groups based on the optimum threshold segmentation of TMB status population. We analyzed the relationship between risk score and TMB. Then, survival analysis between high TMB and low TMB groups was analyzed. The waterfall plots showed the top 20 mutation frequency genes in the high- and low-risk groups by R package “maftools^46^”.

### TME and immune infiltration analysis

ESTIMATE algorithm is applied to estimate stromal and immune cells infiltration in TME. Immune, stromal and ESTIMATE scores of patients from TCGA cohort were obtained via R package “ESTIMATE”. Next, the proportions of 22 immune cells in ccRCC patients were calculated via “CIBERSORT” algorithm^47^. R packages "limma" and "ggpubr" were used to analyze immune infiltration status between the high- and low-risk groups.

### Evaluation of the sensitivity of chemotherapeutic drugs

Drug sensitivity data was downloaded from Genomics of Drug Sensitivity in Cancer (GDSC) database (https://www.cancerrxgene.org/). The IC50 of chemotherapy drugs was calculated via R package “pRRophetic” to evaluate the sensitivity of ccRCC samples to 8 chemotherapeutic drugs^48^. Common anti-tumor chemotherapeutic drugs such as Docetaxel, Gefitinib, Methotrexate, Paclitaxel, Sorafenib, Sunitinib, Vinblastine and Vorinostat were included into analysis. IC50 difference between the high- and low-risk groups was compared using Wilcoxon signed-rank test.

### Quantitative real-time PCR (qRT-PCR)

70 ccRCC samples were obtained from FAHWMU, which was accepted by the Ethics Committee of FAHWMU. Informed consents were signed by all the patients/participants in this study. The mRNA expressions were examined by qRT-PCR. Firstly, TRIzol reagent was supplied to extract the total RNA from tissues and cells. Then, TOROIVD qRT-PCR Master Mix was used to complete the reverse transcription of mRNA to cDNA. Glyceraldehyde-3-phosphate dehydrogenase (GAPDH) served as an internal reference control. The 7500 rapid quantitative PCR system was used to perform real-time PCR using SYBR Green master mix. The Ct values of genes were recorded, and the relative expressions of mRNA were calculated with the formula 2^−ΔCt^. Primer sequences of PAFAH2 and GAPDH were listed in Table S4.

### Cell culture

The human normal kidney cell line (HK-2) and human renal cancer cell line (786-O and ACHN) were purchased from ATCC. HK-2 and ACHN were cultured in MEM medium with 10% fetal bovine serum (FBS) and 1% antibiotics. 786-O was cultured in RPMI-1640 medium with 10% FBS and 1% antibiotics. 37 °C incubator was used for cell culture with 5% CO_2_.

### Cell transfection

In this study, PAFAH2 small interfering RNA (si-PAFAH2) was used to inhibit PAFAH2 in cells and negative control RNA (si-NC) was used as the control. In brief, 786-O cells were cultured in a 6-well plate with 1 × 10^4^ cells per well. In brief, cells were transfected with si-NC, si-PAFAH2#1 or si-PAFAH2#2. The primer sequences of si-NC, si-PAFAH2#1 and si-PAFAH2#2 were listed in Table S4.

### Cell migration assays

Transwell chambers were used for cell migration assays. Briefly, 5 × 10^4^ 786-O cells in 200 μl 1640 medium without FBS were cultured in Transwell filter membrane chambers. A medium containing 20% FBS was added into the lower transwell chambers. Then, cells were cultured in a 37 °C incubator with 5% CO_2_ for 36 h. Subsequently, 4% paraformaldehyde was used for the fix of transwell chambers. After transwell chambers were dried, 0.1% crystal violet solution was used for the cell staining.

### Cell proliferation assays

In accordance with the manufacturer's instructions, we performed cell proliferation assays using Cell Counting Kit-8 (CCK8) (Dojindo, Japan). 786-O cells were planted at a density of 2 × 10^3^/100 μl per well in 96-well plate. Then, 10 μl CCK-8 reagent was used to treat cells for 2 h. Next, the optical density (OD)_450_ values of each well were measured by a microplate reader.

### Statistical analysis

R software and GraphPad Prism were used for all statistical analyses in this study. All R packages were from R programming language. Wilcoxon test and oneway ANOVA were used for comparison analysis. *P* < 0.05 was considered as statistically significant.

### Ethics approval and consent to participate

The studies involving human participants were reviewed and approved by the Human Research Ethics Committee in the First Affiliated Hospital of Wenzhou Medical University. The patients/participants provided their written informed consent to participate in this study. All authors had participated in the collection of clinical information. All methods of this study were carried out in accordance with the Declaration of Helsinki.

## Supplementary Information

Supplementary Information.

## Data Availability

Publicly available datasets were analyzed in this study. This data can be found here: https://portal.gdc.cancer.gov/ (TCGA-ccRCC) and https://www.ncbi.nlm.nih.gov/geo/ (GEO- GSE22541).

## References

[1] Rini BI, Rathmell WK, Godley P (2008). Renal cell carcinoma. Curr. Opin. Oncol..

[2] Ljungberg B, Bensalah K, Canfield S, Dabestani S, Hofmann F, Hora M, Kuczyk MA, Lam T, Marconi L, Merseburger AS (2015). EAU guidelines on renal cell carcinoma: 2014 update. Eur. Urol..

[3] Hakimi AA, Voss MH, Kuo F, Sanchez A, Liu M, Nixon BG, Vuong L, Ostrovnaya I, Chen YB, Reuter V (2019). Transcriptomic profiling of the tumor microenvironment reveals distinct subgroups of clear cell renal cell cancer: Data from a randomized phase III trial. Cancer Discov..

[4] Posadas EM, Limvorasak S, Figlin RA (2017). Targeted therapies for renal cell carcinoma. Nat. Rev. Nephrol..

[5] Tucker MD, Rini BI (2020). Predicting Response to Immunotherapy in Metastatic Renal Cell Carcinoma. Cancers Basel.

[6] Kroemer G, Pouyssegur J (2008). Tumor cell metabolism: Cancer's Achilles' heel. Cancer Cell.

[7] Wettersten HI, Aboud OA, Lara PN, Weiss RH (2017). Metabolic reprogramming in clear cell renal cell carcinoma. Nat. Rev. Nephrol..

[8] Wettersten HI, Hakimi AA, Morin D, Bianchi C, Johnstone ME, Donohoe DR, Trott JF, Aboud OA, Stirdivant S, Neri B (2015). Grade-dependent metabolic reprogramming in kidney cancer revealed by combined proteomics and metabolomics analysis. Cancer Res..

[9] Gebhard RL, Clayman RV, Prigge WF, Figenshau R, Staley NA, Reesey C, Bear A (1987). Abnormal cholesterol metabolism in renal clear cell carcinoma. J. Lipid Res..

[10] Lai Y, Tang F, Huang Y, He C, Chen C, Zhao J, Wu W, He Z (2021). The tumour microenvironment and metabolism in renal cell carcinoma targeted or immune therapy. J. Cell Physiol..

[11] Kim MC, Jin Z, Kolb R, Borcherding N, Chatzkel JA, Falzarano SM, Zhang W (2021). Updates on Immunotherapy and Immune Landscape in Renal Clear Cell Carcinoma. Cancers Basel.

[12] Liu X, Zhang W, Wang H, Zhu L, Xu K (2021). Decreased expression of ACADSB predicts poor prognosis in clear cell renal cell carcinoma. Front Oncol..

[13] Xiao H, Chen P, Zeng G, Xu D, Wang X, Zhang X (2019). Three novel hub genes and their clinical significance in clear cell renal cell carcinoma. J. Cancer.

[14] Jiang H, Chen H, Wan P, Chen N (2021). Decreased expression of HADH is related to poor prognosis and immune infiltration in kidney renal clear cell carcinoma. Genomics.

[15] Weijin F, Zhibin X, Shengfeng Z, Xiaoli Y, Qijian D, Jiayi L, Qiumei L, Yilong C, Hua M, Deyun L, Jiwen C (2019). The clinical significance of PYCR1 expression in renal cell carcinoma. Med. Baltim..

[16] Zhu X, Xu A, Zhang Y, Huo N, Cong R, Ma L, Chu Z, Tang Z, Kang X, Xian S, Xu X (2020). ITPKA1 promotes growth, migration and invasion of renal cell carcinoma via activation of mTOR signaling pathway. Onco. Targets Ther..

[17] Sanchez DJ, Simon MC (2018). Genetic and metabolic hallmarks of clear cell renal cell carcinoma. Biochim. Biophys. Acta Rev. Cancer.

[18] Hsieh JJ, Purdue MP, Signoretti S, Swanton C, Albiges L, Schmidinger M, Heng DY, Larkin J, Ficarra V (2017). Renal cell carcinoma. Nat. Rev. Dis. Primers.

[19] Yu S, Dai J, Ma M, Xu T, Kong Y, Cui C, Chi Z, Si L, Tang H, Yang L (2019). RBCK1 promotes p53 degradation via ubiquitination in renal cell carcinoma. Cell Death Dis..

[20] Lu D, Yang Z, Xia Q, Gao S, Sun S, Luo X, Li Z, Zhang X, Li X (2020). ACADSB regulates ferroptosis and affects the migration, invasion, and proliferation of colorectal cancer cells. Cell Biol. Int..

[21] Ma APY, Yeung CLS, Tey SK, Mao X, Wong SWK, Ng TH, Ko FCF, Kwong EML, Tang AHN, Ng IO (2021). Suppression of ACADM-mediated fatty acid oxidation promotes hepatocellular carcinoma via aberrant CAV1/SREBP1 signaling. Cancer Res..

[22] Shen C, Song YH, Xie Y, Wang X, Wang Y, Wang C, Liu S, Xue SL, Li Y, Liu B (2017). Downregulation of HADH promotes gastric cancer progression via Akt signaling pathway. Oncotarget.

[23] Reversade B, Escande-Beillard N, Dimopoulou A, Fischer B, Chng SC, Li Y, Shboul M, Tham PY, Kayserili H, Al-Gazali L (2009). Mutations in PYCR1 cause cutis laxa with progeroid features. Nat. Genet..

[24] Lee LS, Yuen JS, Sim HG (2011). Renal cell carcinoma in young patients is associated with poorer prognosis. Ann. Acad. Med. Singap..

[25] Ke Y, Karki P, Kim J, Son S, Berdyshev E, Bochkov VN, Birukova AA, Birukov KG (2019). Elevated truncated oxidized phospholipids as a factor exacerbating ALI in the aging lungs. FASEB J..

[26] Pardoll DM (2012). The blockade of immune checkpoints in cancer immunotherapy. Nat. Rev. Cancer.

[27] Labriola MK, Zhu J, Gupta RT, McCall S, Jackson J, Kong EF, White JR, Cerqueira G, Gerding K, Simmons JK (2020). Characterization of tumor mutation burden, PD-L1 and DNA repair genes to assess relationship to immune checkpoint inhibitors response in metastatic renal cell carcinoma. J. Immunother. Cancer.

[28] Chalmers ZR, Connelly CF, Fabrizio D, Gay L, Ali SM, Ennis R, Schrock A, Campbell B, Shlien A, Chmielecki J (2017). Analysis of 100,000 human cancer genomes reveals the landscape of tumor mutational burden. Genome Med..

[29] Galuppini F, Dal Pozzo CA, Deckert J, Loupakis F, Fassan M, Baffa R (2019). Tumor mutation burden: From comprehensive mutational screening to the clinic. Cancer Cell Int..

[30] Bauer DE, Harris MH, Plas DR, Lum JJ, Hammerman PS, Rathmell JC, Riley JL, Thompson CB (2004). Cytokine stimulation of aerobic glycolysis in hematopoietic cells exceeds proliferative demand. FASEB J..

[31] Fox CJ, Hammerman PS, Thompson CB (2005). Fuel feeds function: Energy metabolism and the T-cell response. Nat. Rev. Immunol..

[32] Andrejeva G, Rathmell JC (2017). Similarities and distinctions of cancer and immune metabolism in inflammation and tumors. Cell Metab..

[33] Daurkin I, Eruslanov E, Stoffs T, Perrin GQ, Algood C, Gilbert SM, Rosser CJ, Su LM, Vieweg J, Kusmartsev S (2011). Tumor-associated macrophages mediate immunosuppression in the renal cancer microenvironment by activating the 15-lipoxygenase-2 pathway. Cancer Res..

[34] Han TD, Shang DH, Tian Y (2016). Docetaxel enhances apoptosis and G2/M cell cycle arrest by suppressing mitogen-activated protein kinase signaling in human renal clear cell carcinoma. Genet. Mol. Res..

[35] Vaishampayan U, Flaherty L, Du W, Hussain M (2001). Phase II evaluation of paclitaxel, alpha-interferon, and cis-retinoic acid in advanced renal cell carcinoma. Cancer.

[36] Escudier B, Eisen T, Stadler WM, Szczylik C, Oudard S, Siebels M, Negrier S, Chevreau C, Solska E, Desai AA (2007). Sorafenib in advanced clear-cell renal-cell carcinoma. N. Engl. J. Med..

[37] Mejean A, Ravaud A, Thezenas S, Colas S, Beauval JB, Bensalah K, Geoffrois L, Thiery-Vuillemin A, Cormier L, Lang H (2018). Sunitinib alone or after nephrectomy in metastatic renal-cell carcinoma. N. Engl. J. Med..

[38] Haas NB, Giantonio BJ, Litwin S, Minniti CJ, Fox S, Yeslow G, Reilly R, Nahum K, Greenberg R, Halbherr T, Hudes GR (2003). Vinblastine and estramustine phosphate in metastatic renal cell carcinoma: A phase II trial of the fox chase network. Cancer.

[39] Zibelman M, Wong YN, Devarajan K, Malizzia L, Corrigan A, Olszanski AJ, Denlinger CS, Roethke SK, Tetzlaff CH, Plimack ER (2015). Phase I study of the mTOR inhibitor ridaforolimus and the HDAC inhibitor vorinostat in advanced renal cell carcinoma and other solid tumors. Invest. New Drugs.

[40] Xu W, Liu Z, Ren H, Peng X, Wu A, Ma D, Liu G, Liu L (2020). Twenty metabolic genes based signature predicts survival of glioma patients. J. Cancer.

[41] Wu Y, Wei X, Feng H, Hu B, Liu B, Luan Y, Ruan Y, Liu X, Liu Z, Wang S (2020). An eleven metabolic gene signature-based prognostic model for clear cell renal cell carcinoma. Aging Albany NY.

[42] Ritchie ME, Phipson B, Wu D, Hu Y, Law CW, Shi W, Smyth GK (2015). Limma powers differential expression analyses for RNA-sequencing and microarray studies. Nucleic Acids Res..

[43] Langfelder P, Horvath S (2008). WGCNA: An R package for weighted correlation network analysis. BMC Bioinform..

[44] Yu G, Wang LG, Han Y, He QY (2012). clusterProfiler: An R package for comparing biological themes among gene clusters. OMICS.

[45] Chan TA, Yarchoan M, Jaffee E, Swanton C, Quezada SA, Stenzinger A, Peters S (2019). Development of tumor mutation burden as an immunotherapy biomarker: Utility for the oncology clinic. Ann. Oncol..

[46] Mayakonda A, Lin DC, Assenov Y, Plass C, Koeffler HP (2018). Maftools: efficient and comprehensive analysis of somatic variants in cancer. Genome Res..

[47] Newman AM, Liu CL, Green MR, Gentles AJ, Feng W, Xu Y, Hoang CD, Diehn M, Alizadeh AA (2015). Robust enumeration of cell subsets from tissue expression profiles. Nat. Methods.

[48] Geeleher P, Cox N, Huang RS (2014). pRRophetic: An R package for prediction of clinical chemotherapeutic response from tumor gene expression levels. PLoS ONE.

