# Supplementary material for: Better prediction of clinical outcome in clear cell renal cell carcinoma based on a 6 metabolism-related gene signature
Source: Sci Rep. 2023 Jul 17;13:11490. doi: 10.1038/s41598-023-38380-7 (PMC10352344; doi:10.1038/s41598-023-38380-7)

**Table S1** The data of 318 DEMRGs.

| DEMRG | logFC | pValue | FDR |
| --- | --- | --- | --- |
| MBOAT2 | -1.56334 | 8.02E-36 | 1.45E-34 |
| LIPG | -1.30904 | 2.55E-23 | 9.48E-23 |
| DGKZ | 1.304841 | 1.20E-34 | 1.58E-33 |
| DGKD | 2.148665 | 4.24E-37 | 1.24E-35 |
| DGKH | 1.006381 | 3.91E-18 | 1.04E-17 |
| GK | -1.4119 | 1.11E-08 | 1.81E-08 |
| TKFC | -1.19143 | 2.19E-25 | 9.48E-25 |
| GLYCTK | -1.03308 | 4.81E-06 | 6.82E-06 |
| LPL | -1.20717 | 2.99E-09 | 5.04E-09 |
| ALDH1B1 | -1.21988 | 2.00E-17 | 5.04E-17 |
| DGKB | -1.33802 | 6.46E-11 | 1.16E-10 |
| DGKA | 1.138896 | 7.12E-20 | 2.12E-19 |
| DGKI | 1.66586 | 0.007375 | 0.008688 |
| GPAT3 | -2.50872 | 3.30E-35 | 5.08E-34 |
| PNPLA3 | -1.50716 | 0.002809 | 0.003404 |
| CEL | -2.88884 | 5.23E-35 | 7.63E-34 |
| GPAT2 | 1.389278 | 5.72E-16 | 1.33E-15 |
| GPD1 | -1.62188 | 3.88E-05 | 5.13E-05 |
| GPD1L | -1.49512 | 1.23E-35 | 2.09E-34 |
| LPCAT1 | 2.52377 | 3.13E-36 | 6.02E-35 |
| ACHE | 1.287893 | 0.003934 | 0.004706 |
| PLA2G3 | -2.13552 | 4.10E-43 | 3.47E-40 |
| JMJD7-PLA2G4B | 1.602351 | 1.14E-13 | 2.33E-13 |
| PLA2G6 | 1.293639 | 3.84E-11 | 6.99E-11 |
| PLA2G10 | 1.347128 | 5.08E-11 | 9.18E-11 |
| ETNK2 | -1.65433 | 1.01E-21 | 3.33E-21 |
| PLA2G4A | -1.59552 | 8.22E-26 | 3.70E-25 |
| PLA2G5 | 2.785784 | 4.29E-26 | 2.02E-25 |
| PLA2G4B | 1.902785 | 4.61E-13 | 9.09E-13 |
| PCYT2 | -1.32896 | 1.14E-30 | 7.78E-30 |
| CDS1 | -1.41979 | 1.22E-32 | 1.10E-31 |
| PLA2G1B | 1.177367 | 2.04E-05 | 2.76E-05 |
| TAZ | 1.100045 | 2.19E-28 | 1.20E-27 |
| PLA2G2C | 1.326178 | 1.97E-05 | 2.68E-05 |
| PLA2G2D | 3.46624 | 2.11E-19 | 6.11E-19 |
| CHKB | 1.72254 | 7.36E-20 | 2.17E-19 |
| LCAT | 1.810591 | 4.20E-23 | 1.53E-22 |
| ENPP2 | 1.597591 | 4.69E-11 | 8.50E-11 |
| ENPP6 | -4.90889 | 4.54E-35 | 6.75E-34 |
| PAFAH2 | -1.0413 | 2.89E-39 | 1.83E-37 |
| PLA2G7 | 3.638496 | 1.20E-36 | 3.00E-35 |
| EPHX2 | -1.32956 | 1.70E-26 | 8.07E-26 |
| ALOX5 | 2.080095 | 3.05E-25 | 1.29E-24 |
| CYP4A11 | -1.52707 | 2.57E-07 | 3.88E-07 |
| CYP2J2 | 5.182943 | 1.17E-30 | 7.91E-30 |
| ALOX12B | 1.965209 | 1.93E-16 | 4.61E-16 |
| CYP4F2 | -4.96912 | 4.28E-17 | 1.06E-16 |
| ALOX15B | 4.274309 | 2.52E-34 | 3.19E-33 |
| PTGS2 | -1.19252 | 2.04E-14 | 4.31E-14 |
| ALOX12 | 1.273441 | 2.06E-24 | 8.08E-24 |
| CYP2C8 | 1.905788 | 6.34E-11 | 1.14E-10 |
| CBR1 | -1.00708 | 1.83E-29 | 1.11E-28 |
| CYP2B6 | -4.60305 | 1.70E-36 | 3.79E-35 |
| CBR3 | 1.197959 | 4.62E-18 | 1.22E-17 |
| CYP4A22 | -1.73182 | 1.63E-07 | 2.50E-07 |
| GPX2 | -1.2493 | 4.76E-28 | 2.44E-27 |
| GPX3 | -1.56049 | 5.82E-05 | 7.65E-05 |
| CYP4F3 | -2.20682 | 3.06E-11 | 5.64E-11 |
| LTC4S | 1.153699 | 1.72E-07 | 2.63E-07 |
| GGT6 | -4.92803 | 3.39E-40 | 4.79E-38 |
| PTGDS | -1.24354 | 3.82E-14 | 7.99E-14 |
| TBXAS1 | 1.817103 | 8.93E-36 | 1.58E-34 |
| CYP3A5 | 2.162583 | 1.17E-21 | 3.81E-21 |
| CYP3A4 | -1.33932 | 1.47E-05 | 2.03E-05 |
| CYP3A43 | 1.951412 | 3.39E-11 | 6.21E-11 |
| GAL3ST1 | 2.922702 | 2.39E-30 | 1.56E-29 |
| SPHK2 | -1.16327 | 1.29E-34 | 1.69E-33 |
| DEGS2 | -3.08332 | 9.17E-30 | 5.67E-29 |
| SPHK1 | 1.66535 | 6.24E-19 | 1.75E-18 |
| DEGS1 | 1.247658 | 3.85E-31 | 2.70E-30 |
| SGPP1 | -1.25687 | 2.51E-38 | 1.12E-36 |
| NEU4 | -1.62438 | 2.46E-21 | 7.87E-21 |
| UGT8 | -1.77731 | 9.39E-32 | 7.04E-31 |
| ACP4 | 1.626657 | 1.67E-12 | 3.21E-12 |
| ENPP3 | 4.668276 | 3.06E-32 | 2.54E-31 |
| ENPP1 | -1.07745 | 5.50E-23 | 1.99E-22 |
| NNT | -1.26424 | 2.96E-33 | 2.85E-32 |
| AOX1 | -1.38682 | 5.09E-08 | 8.05E-08 |
| CD38 | 1.907797 | 7.46E-18 | 1.94E-17 |
| NADSYN1 | 1.079299 | 1.29E-33 | 1.39E-32 |
| NT5C1A | -4.12102 | 3.76E-23 | 1.38E-22 |
| QPRT | -1.15466 | 7.63E-22 | 2.54E-21 |
| PNP | -1.43678 | 3.13E-35 | 4.91E-34 |
| NMNAT2 | 1.656458 | 0.000558 | 0.000706 |
| NNMT | 3.873519 | 9.25E-36 | 1.60E-34 |
| FBP2 | -1.47116 | 5.94E-24 | 2.28E-23 |
| GMDS | -1.01717 | 8.07E-32 | 6.27E-31 |
| PFKFB4 | 2.401638 | 2.61E-34 | 3.25E-33 |
| PFKFB3 | -1.2901 | 0.039439 | 0.044186 |
| PFKFB2 | -1.71693 | 9.19E-35 | 1.26E-33 |
| PFKFB1 | -1.0814 | 9.51E-19 | 2.62E-18 |
| FBP1 | -2.26581 | 4.77E-26 | 2.23E-25 |
| HK2 | 3.423749 | 2.37E-36 | 4.89E-35 |
| HK3 | 2.608662 | 6.24E-34 | 7.24E-33 |
| ALDOA | 1.075525 | 2.79E-32 | 2.36E-31 |
| ALDOC | 2.257622 | 4.42E-25 | 1.84E-24 |
| ALDOB | -4.69342 | 3.15E-20 | 9.48E-20 |
| SORD | -2.11494 | 1.93E-24 | 7.60E-24 |
| PFKP | 1.966743 | 2.81E-33 | 2.73E-32 |
| G6PC2 | 1.377279 | 0.003838 | 0.004598 |
| LCT | 2.448635 | 9.06E-09 | 1.48E-08 |
| G6PC | -3.40727 | 4.61E-13 | 9.09E-13 |
| UGT1A10 | 2.812648 | 0.001148 | 0.001434 |
| UGT1A8 | 1.719091 | 0.02994 | 0.033767 |
| UGT1A7 | 2.10778 | 0.042631 | 0.047574 |
| UGT1A5 | 4.138789 | 3.55E-08 | 5.65E-08 |
| MIOX | -2.21866 | 2.88E-06 | 4.17E-06 |
| UGT2A1 | -4.54349 | 2.89E-27 | 1.42E-26 |
| UGT2B11 | 1.187962 | 0.001372 | 0.001696 |
| UGT2B7 | -1.1984 | 0.022277 | 0.025532 |
| UGT1A4 | 4.542383 | 1.35E-10 | 2.39E-10 |
| UGT1A1 | 2.632168 | 7.31E-17 | 1.79E-16 |
| UGT2B17 | 2.012027 | 3.30E-06 | 4.75E-06 |
| UGT1A3 | 3.9105 | 3.13E-22 | 1.08E-21 |
| ACADSB | -1.94561 | 2.12E-35 | 3.39E-34 |
| ACADM | -1.46745 | 2.02E-33 | 2.07E-32 |
| ACAT1 | -1.20254 | 4.68E-28 | 2.42E-27 |
| HADH | -1.75641 | 6.35E-40 | 7.69E-38 |
| ADH6 | -2.55175 | 1.09E-09 | 1.86E-09 |
| ACSL6 | -2.1055 | 9.22E-34 | 1.03E-32 |
| ADH1B | -2.72464 | 7.11E-34 | 8.13E-33 |
| ADH1C | -3.46341 | 3.42E-39 | 1.83E-37 |
| ECHS1 | -1.30875 | 2.96E-25 | 1.26E-24 |
| GCDH | -1.02945 | 1.44E-26 | 6.91E-26 |
| ACAA1 | -1.5698 | 7.74E-32 | 6.07E-31 |
| CPT1B | 1.880884 | 2.94E-10 | 5.15E-10 |
| ECI2 | -1.0429 | 2.68E-30 | 1.73E-29 |
| ACSL4 | -1.51224 | 2.99E-38 | 1.27E-36 |
| POLR2F | 1.718997 | 8.50E-24 | 3.22E-23 |
| NME1-NME2 | 1.023155 | 4.55E-17 | 1.12E-16 |
| GUCY2D | 1.498745 | 0.009042 | 0.010593 |
| NME3 | 1.007036 | 1.02E-21 | 3.34E-21 |
| PDE6C | 1.792547 | 3.69E-18 | 9.87E-18 |
| PDE6A | 2.025048 | 2.87E-11 | 5.30E-11 |
| GMPR | -2.39457 | 3.39E-35 | 5.13E-34 |
| ADCY2 | 2.630011 | 2.85E-06 | 4.13E-06 |
| ADA | 2.195584 | 5.15E-34 | 6.23E-33 |
| ENTPD8 | -1.72451 | 2.62E-25 | 1.12E-24 |
| POLE2 | 1.36313 | 2.61E-31 | 1.85E-30 |
| PDE1B | 1.658485 | 3.03E-28 | 1.63E-27 |
| PDE6B | 1.003001 | 3.49E-13 | 6.92E-13 |
| PDE7A | 1.231877 | 4.09E-22 | 1.39E-21 |
| PDE6G | -1.14816 | 1.02E-19 | 2.99E-19 |
| ADCY8 | 3.208978 | 0.002645 | 0.00321 |
| AK7 | -1.49986 | 1.27E-31 | 9.44E-31 |
| ADCY7 | 1.995563 | 1.74E-24 | 6.96E-24 |
| ADCY10 | 1.952159 | 4.28E-19 | 1.22E-18 |
| PDE1A | -2.8883 | 2.89E-39 | 1.83E-37 |
| POLR2J3 | 1.009699 | 2.73E-15 | 6.10E-15 |
| POLR3B | -1.22103 | 5.94E-37 | 1.62E-35 |
| GUCY1B1 | 1.209497 | 3.37E-29 | 1.98E-28 |
| ADK | -1.18161 | 3.17E-37 | 9.94E-36 |
| RRM2 | 2.123929 | 2.81E-32 | 2.36E-31 |
| PDE8B | -1.05687 | 4.57E-21 | 1.44E-20 |
| ENTPD2 | 1.263635 | 1.33E-11 | 2.48E-11 |
| ENTPD3 | -3.22155 | 9.12E-38 | 3.36E-36 |
| ENTPD1 | 1.024025 | 3.97E-28 | 2.09E-27 |
| TYMS | 2.116513 | 1.43E-36 | 3.36E-35 |
| UPP2 | -4.25279 | 7.74E-29 | 4.40E-28 |
| DPYS | -1.36472 | 0.002108 | 0.002572 |
| TYMP | 2.944263 | 4.69E-37 | 1.32E-35 |
| UPP1 | 1.010027 | 1.52E-15 | 3.46E-15 |
| AK3 | -1.46617 | 1.50E-39 | 1.27E-37 |
| AGXT2 | -1.11895 | 0.005502 | 0.006536 |
| ABAT | -2.97889 | 2.05E-25 | 8.96E-25 |
| IL4I1 | 2.088999 | 2.42E-27 | 1.20E-26 |
| GPT2 | -1.12316 | 2.93E-18 | 7.87E-18 |
| GAD2 | 3.574279 | 2.20E-12 | 4.23E-12 |
| GOT2 | -1.10804 | 5.42E-35 | 7.65E-34 |
| AGXT | -2.1439 | 1.16E-12 | 2.26E-12 |
| GOT1 | -1.05655 | 4.68E-28 | 2.42E-27 |
| ALDH4A1 | -2.04074 | 5.93E-19 | 1.66E-18 |
| ALDH5A1 | -1.03649 | 4.30E-30 | 2.74E-29 |
| ASS1 | -2.65627 | 2.32E-15 | 5.22E-15 |
| ALAS1 | -1.03442 | 1.16E-33 | 1.26E-32 |
| GATM | -2.07395 | 4.09E-08 | 6.47E-08 |
| SDS | 4.39474 | 1.86E-39 | 1.43E-37 |
| GCAT | -1.09409 | 3.77E-23 | 1.38E-22 |
| PHGDH | -1.35606 | 6.45E-26 | 2.99E-25 |
| SHMT2 | 1.978988 | 1.22E-37 | 4.32E-36 |
| PSAT1 | -2.49609 | 4.15E-24 | 1.60E-23 |
| BHMT | -1.18451 | 0.006948 | 0.00822 |
| GLDC | -2.32025 | 2.37E-36 | 4.89E-35 |
| SARDH | -1.18201 | 5.11E-08 | 8.07E-08 |
| AMT | -1.06667 | 2.89E-28 | 1.56E-27 |
| CHDH | -1.24036 | 8.88E-22 | 2.94E-21 |
| CBS | -1.09472 | 3.51E-15 | 7.81E-15 |
| CTH | -1.85663 | 1.54E-37 | 5.21E-36 |
| PIPOX | -2.91094 | 8.50E-10 | 1.46E-09 |
| DAO | -3.18305 | 1.16E-11 | 2.17E-11 |
| LDHC | -1.44796 | 1.40E-13 | 2.82E-13 |
| LDHB | -1.55261 | 8.55E-39 | 4.26E-37 |
| CDO1 | -1.63778 | 9.38E-34 | 1.03E-32 |
| LDHA | 1.656938 | 7.71E-35 | 1.07E-33 |
| DNMT3L | -2.81737 | 8.98E-18 | 2.32E-17 |
| ARG1 | 2.319345 | 1.47E-12 | 2.84E-12 |
| ARG2 | -1.96062 | 2.69E-27 | 1.33E-26 |
| CKMT1A | -1.13752 | 6.11E-28 | 3.08E-27 |
| P4HA2 | 1.313876 | 6.12E-25 | 2.53E-24 |
| NOS2 | 1.019922 | 0.002424 | 0.002954 |
| NOS1 | -4.29274 | 1.54E-29 | 9.45E-29 |
| P4HA1 | 1.711656 | 2.77E-34 | 3.41E-33 |
| AGMAT | -2.33889 | 5.88E-08 | 9.17E-08 |
| PYCR1 | 1.67037 | 2.67E-11 | 4.96E-11 |
| P4HA3 | 2.773836 | 6.55E-29 | 3.77E-28 |
| PRODH2 | -2.16509 | 0.000158 | 0.000204 |
| OAT | -1.18122 | 1.11E-36 | 2.85E-35 |
| CKM | -2.79631 | 1.68E-16 | 4.05E-16 |
| ACY1 | -2.12503 | 2.08E-14 | 4.37E-14 |
| SAT2 | -1.1092 | 5.14E-22 | 1.73E-21 |
| CKMT1B | -1.14452 | 1.98E-29 | 1.19E-28 |
| CNDP1 | -1.16884 | 1.94E-10 | 3.42E-10 |
| HAL | 2.302267 | 3.75E-16 | 8.78E-16 |
| DDC | -1.60955 | 1.80E-05 | 2.46E-05 |
| ALDH1A3 | -1.49631 | 1.87E-05 | 2.55E-05 |
| ALDH3B2 | -3.88567 | 1.33E-39 | 1.27E-37 |
| UROC1 | 2.044167 | 1.74E-09 | 2.94E-09 |
| FTCD | -1.15498 | 7.69E-05 | 0.000101 |
| HDC | 1.91176 | 1.06E-17 | 2.72E-17 |
| HPD | -4.84664 | 1.39E-24 | 5.62E-24 |
| TYRP1 | -5.22297 | 1.50E-39 | 1.27E-37 |
| MIF | 1.146356 | 1.44E-20 | 4.43E-20 |
| PNMT | -2.59866 | 8.69E-32 | 6.57E-31 |
| TPO | 1.979732 | 9.09E-15 | 1.96E-14 |
| TH | -2.12442 | 1.02E-24 | 4.20E-24 |
| PAH | -2.77384 | 5.88E-12 | 1.11E-11 |
| AANAT | 2.688111 | 2.01E-29 | 1.20E-28 |
| IDO1 | 3.338151 | 2.43E-36 | 4.91E-35 |
| CAT | -1.27406 | 2.08E-33 | 2.09E-32 |
| AFMID | -1.02702 | 9.13E-26 | 4.09E-25 |
| CYP1A1 | -3.07755 | 4.58E-28 | 2.39E-27 |
| CYP1B1 | -1.16358 | 1.52E-23 | 5.68E-23 |
| ASMT | 2.093637 | 2.93E-16 | 6.96E-16 |
| OGDHL | -1.98543 | 1.51E-30 | 1.01E-29 |
| TDO2 | 2.047606 | 3.02E-08 | 4.81E-08 |
| HIBCH | -1.1995 | 3.47E-20 | 1.04E-19 |
| CSAD | 1.584299 | 1.24E-13 | 2.52E-13 |
| BAAT | 5.57481 | 2.55E-11 | 4.73E-11 |
| GSTM3 | -3.33121 | 4.21E-41 | 1.19E-38 |
| GSTM5 | -1.26156 | 2.92E-17 | 7.31E-17 |
| IDH2 | -1.12764 | 7.68E-26 | 3.52E-25 |
| GSTO2 | -1.80807 | 1.97E-33 | 2.05E-32 |
| AMY2B | 1.807397 | 2.31E-14 | 4.85E-14 |
| UXS1 | -1.45877 | 1.02E-34 | 1.37E-33 |
| TREH | -2.24246 | 2.55E-18 | 6.90E-18 |
| PYGL | 2.023632 | 1.28E-35 | 2.12E-34 |
| CHIT1 | 4.971159 | 1.23E-21 | 3.98E-21 |
| CHIA | -1.18145 | 2.98E-22 | 1.03E-21 |
| PLCB2 | 2.162994 | 4.12E-31 | 2.86E-30 |
| PLCB4 | 1.083929 | 4.37E-06 | 6.22E-06 |
| PIP5K1B | -1.66583 | 6.40E-33 | 6.02E-32 |
| INPP5J | -2.05391 | 3.99E-36 | 7.34E-35 |
| ITPKA | 3.860543 | 2.01E-27 | 1.00E-26 |
| ALDH6A1 | -2.9566 | 7.95E-34 | 8.98E-33 |
| PIK3C2G | -3.92695 | 2.84E-40 | 4.79E-38 |
| PIP4K2C | -1.41972 | 1.01E-40 | 2.13E-38 |
| PLCZ1 | -1.49109 | 1.98E-33 | 2.05E-32 |
| PIK3CG | 1.282382 | 1.84E-14 | 3.90E-14 |
| PCK2 | -2.21622 | 1.11E-09 | 1.89E-09 |
| PDHB | -1.17116 | 3.87E-37 | 1.17E-35 |
| PCK1 | -2.74061 | 3.76E-19 | 1.08E-18 |
| PDHA1 | -1.17684 | 1.81E-35 | 2.94E-34 |
| LDHD | -2.27814 | 1.43E-31 | 1.05E-30 |
| PC | -1.37343 | 6.36E-15 | 1.39E-14 |
| ACOT12 | -4.31291 | 1.76E-41 | 7.45E-39 |
| ACO1 | -1.05468 | 1.86E-24 | 7.40E-24 |
| MTHFD2 | 1.00668 | 1.55E-17 | 3.94E-17 |
| HAO1 | -1.96387 | 2.43E-15 | 5.46E-15 |
| HAO2 | -2.3816 | 3.14E-10 | 5.48E-10 |
| PCCB | -1.68098 | 7.18E-37 | 1.90E-35 |
| PCCA | -1.42445 | 1.57E-32 | 1.40E-31 |
| SUCLG1 | -1.73339 | 1.47E-36 | 3.36E-35 |
| SUCLA2 | -1.01012 | 2.86E-36 | 5.63E-35 |
| SUCLG2 | -1.27827 | 1.34E-36 | 3.25E-35 |
| OXCT1 | -1.26732 | 4.91E-33 | 4.67E-32 |
| BDH1 | -1.89703 | 5.52E-34 | 6.59E-33 |
| ACSM3 | -1.38989 | 4.57E-29 | 2.65E-28 |
| ACSM4 | 1.954577 | 1.06E-12 | 2.07E-12 |
| HMGCS2 | -2.6365 | 2.58E-31 | 1.85E-30 |
| ACSM5 | 1.032577 | 4.07E-11 | 7.37E-11 |
| L2HGDH | -1.35881 | 1.76E-30 | 1.17E-29 |
| RPE65 | 2.750773 | 3.59E-20 | 1.08E-19 |
| RDH8 | -2.87055 | 5.55E-27 | 2.70E-26 |
| BCO1 | 1.595099 | 1.91E-20 | 5.82E-20 |
| RDH10 | -1.67081 | 2.05E-36 | 4.45E-35 |
| CYP26A1 | 1.938562 | 0.013117 | 0.015199 |
| CYP26B1 | -1.60646 | 1.39E-25 | 6.10E-25 |
| DHRS9 | 1.921393 | 1.57E-24 | 6.31E-24 |
| RDH12 | -2.01016 | 4.97E-06 | 7.03E-06 |
| LRAT | 2.260052 | 1.50E-18 | 4.10E-18 |
| CYP2A6 | 5.4126 | 6.48E-07 | 9.64E-07 |
| CYP2A7 | 3.932605 | 1.28E-05 | 1.77E-05 |
| ALDH1A2 | -1.69497 | 3.22E-32 | 2.65E-31 |
| RDH16 | 1.805737 | 1.17E-08 | 1.90E-08 |
| RDH5 | 1.304622 | 3.38E-17 | 8.41E-17 |
| ALAD | -1.22653 | 1.69E-32 | 1.49E-31 |
| FECH | -1.94919 | 3.09E-39 | 1.83E-37 |
| HMOX1 | 2.368126 | 1.97E-32 | 1.69E-31 |
| CP | 3.669142 | 1.15E-24 | 4.69E-24 |
| CA6 | 3.069378 | 8.53E-17 | 2.08E-16 |
| CA2 | -1.40275 | 1.75E-32 | 1.53E-31 |
| CA4 | -1.39305 | 1.46E-20 | 4.47E-20 |
| CA8 | -3.36731 | 2.44E-38 | 1.12E-36 |
| CA9 | 5.922497 | 5.35E-38 | 2.16E-36 |
| CA1 | 2.508466 | 1.77E-08 | 2.87E-08 |
| SULT1A3 | 2.445366 | 7.53E-15 | 1.64E-14 |
| CHST11 | 1.779393 | 1.13E-32 | 1.06E-31 |
| SULT2B1 | -3.24599 | 6.37E-13 | 1.25E-12 |
| SUOX | -1.04993 | 6.43E-38 | 2.48E-36 |
| CHST13 | 2.021712 | 5.29E-22 | 1.77E-21 |
| CYP2F1 | 2.995822 | 3.16E-05 | 4.21E-05 |
| AKR1C2 | -1.267 | 0.000734 | 0.000923 |
| AKR1C1 | -1.13681 | 1.97E-08 | 3.17E-08 |
| DHDH | -1.11754 | 0.001773 | 0.002174 |
| CYP2D6 | 1.49667 | 1.65E-06 | 2.41E-06 |
| FMO4 | -1.32243 | 7.17E-20 | 2.12E-19 |
| FMO5 | -2.39771 | 1.80E-37 | 5.86E-36 |
| NAT2 | -1.73763 | 3.30E-29 | 1.95E-28 |

**Table S2** 54 prognostic MRGs selected by univariate cox regression analysis.

| id | HR | HR.95L | HR.95H | pvalue |
| --- | --- | --- | --- | --- |
| GK | 0.668474 | 0.532194 | 0.839652 | 0.000535 |
| ALDH1B1 | 0.739415 | 0.606122 | 0.902019 | 0.002913 |
| GPAT3 | 0.686873 | 0.577579 | 0.816847 | 2.16E-05 |
| GPAT2 | 2.187391 | 1.397991 | 3.42254 | 0.000611 |
| GPD1L | 0.583835 | 0.440654 | 0.773539 | 0.000178 |
| CDS1 | 0.586805 | 0.483356 | 0.712395 | 7.16E-08 |
| PLA2G2C | 4.921838 | 1.582039 | 15.3122 | 0.005921 |
| PAFAH2 | 0.27402 | 0.183952 | 0.408188 | 1.93E-10 |
| EPHX2 | 0.643105 | 0.547948 | 0.754786 | 6.54E-08 |
| CBR3 | 1.377894 | 1.130423 | 1.679543 | 0.001505 |
| PTGDS | 1.131504 | 1.030407 | 1.242521 | 0.009675 |
| SPHK1 | 1.667491 | 1.405793 | 1.977908 | 4.35E-09 |
| UGT8 | 0.707888 | 0.588016 | 0.852198 | 0.000263 |
| NNT | 0.583139 | 0.469669 | 0.724022 | 1.03E-06 |
| PNP | 0.649184 | 0.501002 | 0.841195 | 0.001083 |
| NNMT | 1.200284 | 1.060515 | 1.358473 | 0.003851 |
| ACADSB | 0.492081 | 0.401236 | 0.603493 | 9.77E-12 |
| ACADM | 0.549853 | 0.463141 | 0.652799 | 8.45E-12 |
| ACAT1 | 0.617426 | 0.524556 | 0.72674 | 6.72E-09 |
| HADH | 0.355803 | 0.252967 | 0.500443 | 2.89E-09 |
| ECI2 | 0.460767 | 0.334557 | 0.634588 | 2.09E-06 |
| POLR2F | 10.30001 | 2.066424 | 51.33999 | 0.004433 |
| ADA | 1.443678 | 1.233681 | 1.689422 | 4.69E-06 |
| POLE2 | 2.204779 | 1.524733 | 3.188132 | 2.65E-05 |
| ADCY10 | 2.2014 | 1.330639 | 3.641983 | 0.002126 |
| RRM2 | 1.673718 | 1.403019 | 1.996645 | 1.05E-08 |
| AK3 | 0.57766 | 0.428262 | 0.779175 | 0.000325 |
| ABAT | 0.654104 | 0.517998 | 0.825971 | 0.000362 |
| GAD2 | 11.32794 | 3.581739 | 35.8268 | 3.60E-05 |
| GOT1 | 0.63552 | 0.525274 | 0.768904 | 3.11E-06 |
| ALDH5A1 | 0.702365 | 0.552924 | 0.892197 | 0.003798 |
| CBS | 10.22486 | 3.388447 | 30.85421 | 3.70E-05 |
| PYCR1 | 1.570572 | 1.379062 | 1.788677 | 1.02E-11 |
| P4HA3 | 1.442779 | 1.242424 | 1.675444 | 1.54E-06 |
| CAT | 0.605787 | 0.50946 | 0.720327 | 1.41E-08 |
| CYP1B1 | 1.189838 | 1.066423 | 1.327534 | 0.001865 |
| OGDHL | 0.790018 | 0.704663 | 0.885712 | 5.34E-05 |
| HIBCH | 0.438407 | 0.335052 | 0.573646 | 1.84E-09 |
| PIP5K1B | 0.600328 | 0.427456 | 0.843113 | 0.003232 |
| ITPKA | 1.503698 | 1.34555 | 1.680434 | 6.25E-13 |
| ALDH6A1 | 0.57393 | 0.484818 | 0.679421 | 1.12E-10 |
| LDHD | 0.717902 | 0.610865 | 0.843694 | 5.74E-05 |
| ACO1 | 0.58952 | 0.441337 | 0.787457 | 0.000347 |
| MTHFD2 | 1.703706 | 1.400582 | 2.072433 | 9.81E-08 |
| PCCA | 0.506983 | 0.410866 | 0.625585 | 2.40E-10 |
| SUCLA2 | 0.494183 | 0.396214 | 0.616377 | 4.04E-10 |
| SUCLG2 | 0.556788 | 0.416496 | 0.744336 | 7.71E-05 |
| ACSM4 | 2.967444 | 1.468764 | 5.995328 | 0.002435 |
| L2HGDH | 0.491722 | 0.363728 | 0.664758 | 3.94E-06 |
| RDH8 | 1.587021 | 1.121938 | 2.244897 | 0.009048 |
| FECH | 0.456703 | 0.333401 | 0.625607 | 1.05E-06 |
| CA2 | 0.714283 | 0.614371 | 0.830443 | 1.20E-05 |
| SULT2B1 | 1.503796 | 1.18648 | 1.905976 | 0.000741 |
| AKR1C1 | 0.718806 | 0.604868 | 0.854207 | 0.000177 |

**Table S3** 60 DETFs in ccRCC.

| ID | conMean | treatMean | logFC | pValue | FDR |
| --- | --- | --- | --- | --- | --- |
| ATF3 | 46.77667 | 20.46375 | -1.19272 | 1.17E-09 | 2.20E-09 |
| BACH2 | 0.484031 | 0.23026 | -1.07183 | 2.02E-21 | 8.30E-21 |
| BATF | 0.477197 | 3.85356 | 3.013534 | 6.08E-34 | 1.04E-32 |
| BCL11A | 0.160437 | 0.475625 | 1.567819 | 1.73E-15 | 4.68E-15 |
| CEBPA | 1.504954 | 4.269425 | 1.504322 | 1.49E-18 | 4.95E-18 |
| CEBPB | 9.480237 | 24.1428 | 1.348598 | 2.01E-18 | 6.60E-18 |
| CENPA | 0.131555 | 0.704126 | 2.420167 | 4.13E-33 | 6.03E-32 |
| CIITA | 1.230969 | 3.49899 | 1.507144 | 2.29E-21 | 9.35E-21 |
| E2F1 | 0.613877 | 2.7118 | 2.143229 | 5.71E-35 | 1.24E-33 |
| E2F7 | 0.079399 | 0.336965 | 2.0854 | 1.85E-30 | 1.75E-29 |
| EGR2 | 9.535756 | 4.330145 | -1.13893 | 7.85E-05 | 0.000109 |
| EHF | 19.91222 | 0.52511 | -5.24489 | 1.22E-40 | 2.46E-38 |
| ELF5 | 11.6701 | 0.047323 | -7.94605 | 4.78E-41 | 1.26E-38 |
| EMX1 | 14.03607 | 2.292337 | -2.61425 | 9.41E-41 | 2.16E-38 |
| EOMES | 0.180121 | 2.135259 | 3.567372 | 1.25E-33 | 1.99E-32 |
| EPO | 0.962355 | 8.855615 | 3.201951 | 9.26E-09 | 1.65E-08 |
| ETS1 | 20.11912 | 45.90595 | 1.190114 | 3.37E-21 | 1.36E-20 |
| EZH2 | 0.504125 | 1.857131 | 1.881222 | 4.69E-36 | 1.34E-34 |
| FLI1 | 2.562855 | 6.039957 | 1.236787 | 1.99E-25 | 1.12E-24 |
| FOXM1 | 0.435163 | 2.359438 | 2.438816 | 4.56E-34 | 8.00E-33 |
| FOXP3 | 0.366837 | 1.168879 | 1.671914 | 5.20E-13 | 1.20E-12 |
| GATA2 | 9.248066 | 3.141013 | -1.55792 | 2.79E-30 | 2.58E-29 |
| GATA3 | 25.44815 | 2.867508 | -3.14969 | 4.57E-39 | 3.94E-37 |
| GRHL2 | 4.631645 | 0.333207 | -3.79703 | 1.05E-36 | 3.80E-35 |
| HEY1 | 2.223287 | 6.654565 | 1.58165 | 6.65E-26 | 3.92E-25 |
| HIF1A | 77.61493 | 33.72897 | -1.20235 | 6.60E-24 | 3.27E-23 |
| HOXB7 | 23.16635 | 11.35268 | -1.029 | 4.35E-25 | 2.39E-24 |
| IKZF1 | 0.657617 | 2.852472 | 2.116892 | 1.07E-28 | 8.16E-28 |
| IRF1 | 5.463306 | 12.014 | 1.136871 | 3.27E-20 | 1.22E-19 |
| IRF4 | 0.19247 | 0.781507 | 2.021623 | 8.01E-17 | 2.36E-16 |
| KLF5 | 10.33222 | 3.659709 | -1.49735 | 1.07E-27 | 7.33E-27 |
| LEF1 | 1.373005 | 2.936016 | 1.096523 | 0.021098 | 0.024539 |
| LHX2 | 0.008706 | 0.252055 | 4.855592 | 4.41E-21 | 1.76E-20 |
| LMNB1 | 1.941583 | 4.905947 | 1.337298 | 2.43E-26 | 1.49E-25 |
| LYL1 | 1.134688 | 2.402879 | 1.082468 | 2.88E-21 | 1.17E-20 |
| MEF2B | 0.067171 | 0.2806 | 2.062594 | 6.34E-28 | 4.46E-27 |
| MEF2C | 3.684022 | 9.267533 | 1.330904 | 2.35E-24 | 1.21E-23 |
| MYBL2 | 0.405437 | 2.607002 | 2.684841 | 3.97E-34 | 7.08E-33 |
| MYC | 12.39718 | 35.53526 | 1.51924 | 3.09E-23 | 1.45E-22 |
| NCAPG | 0.21569 | 0.934883 | 2.115827 | 1.97E-34 | 3.74E-33 |
| NR5A2 | 0.501486 | 1.219609 | 1.282136 | 8.90E-20 | 3.23E-19 |
| PBX1 | 7.006296 | 2.743029 | -1.35288 | 6.62E-33 | 9.26E-32 |
| PML | 4.127492 | 9.574672 | 1.213958 | 3.33E-36 | 9.89E-35 |
| POU5F1 | 1.138773 | 12.5255 | 3.459317 | 2.74E-35 | 6.40E-34 |
| PRDM1 | 1.253642 | 6.253365 | 2.318508 | 2.00E-36 | 6.44E-35 |
| RARA | 7.262339 | 15.19484 | 1.065075 | 8.71E-27 | 5.52E-26 |
| RBP2 | 2.037824 | 0.139397 | -3.86976 | 4.08E-36 | 1.18E-34 |
| RFX2 | 0.584436 | 1.650532 | 1.497815 | 1.30E-26 | 8.13E-26 |
| RUNX1 | 1.330868 | 4.475056 | 1.749539 | 1.61E-27 | 1.09E-26 |
| SAP30 | 2.616935 | 14.652 | 2.485148 | 1.83E-38 | 1.26E-36 |
| SPDEF | 0.069527 | 0.433683 | 2.640993 | 6.35E-18 | 2.01E-17 |
| SPIB | 0.110725 | 0.280495 | 1.340994 | 2.77E-11 | 5.74E-11 |
| SREBF2 | 28.61649 | 11.93172 | -1.26205 | 1.42E-35 | 3.57E-34 |
| STAT4 | 0.389246 | 1.453217 | 1.900495 | 1.72E-32 | 2.24E-31 |
| TAL1 | 0.886837 | 1.828429 | 1.043864 | 5.62E-10 | 1.08E-09 |
| TCF21 | 10.25958 | 0.917941 | -3.48243 | 1.15E-39 | 1.38E-37 |
| TFAP2A | 2.712707 | 0.655883 | -2.04822 | 7.17E-34 | 1.21E-32 |
| TFAP2C | 1.775898 | 0.379051 | -2.22808 | 2.65E-24 | 1.36E-23 |
| TP73 | 0.02929 | 0.245782 | 3.068905 | 7.64E-35 | 1.60E-33 |
| VDR | 10.85371 | 3.359952 | -1.69168 | 2.80E-23 | 1.32E-22 |

**Table S4** Primer sequences.

| Gene | Forward sequence | Reverse sequence |
| --- | --- | --- |
| PAFAH2 | 5'- CAGCCCACCAATGAATCGCT -3' | 5'- AAACACTCGCTTACCCGCTG -3' |
| GAPDH | 5'- AAAGCCTGCCGGTGACTAAC -3' | 5'- GCCCAATACGACCAAATCAGA -3' |
| si-NC | 5'- UUCUCCGAACGUGUCACGUTT -3' | 5'- ACGUGACACGUUCGGAGAATT -3' |
| si-PAFAH2#1 | 5'- CUGAGAAAUUCCAGACAAUTT -3' | 5'- AUUGUCUGGAAUUUCUCAGTT -3' |
| si-PAFAH2#2 | 5'- AGACAAAGGACUCUGGAUATT -3' | 5'- UAUCCAGAGUCCUUUGUCUTT -3' |

**Fig.S1** Enrichment and regulatory analysis of 54 prognostic MRGs. GO (a) and KEGG (b) functional enrichment analysis of prognostic MRGs. (c) TF-prognostic MRGs regulatory network; the triangle represents TF, the circle represents prognostic MRGs (red color meant the risk MRGs, green color meant the protective MRGs), the red lines represent the positive correlation and the green lines represent the negative correlation.


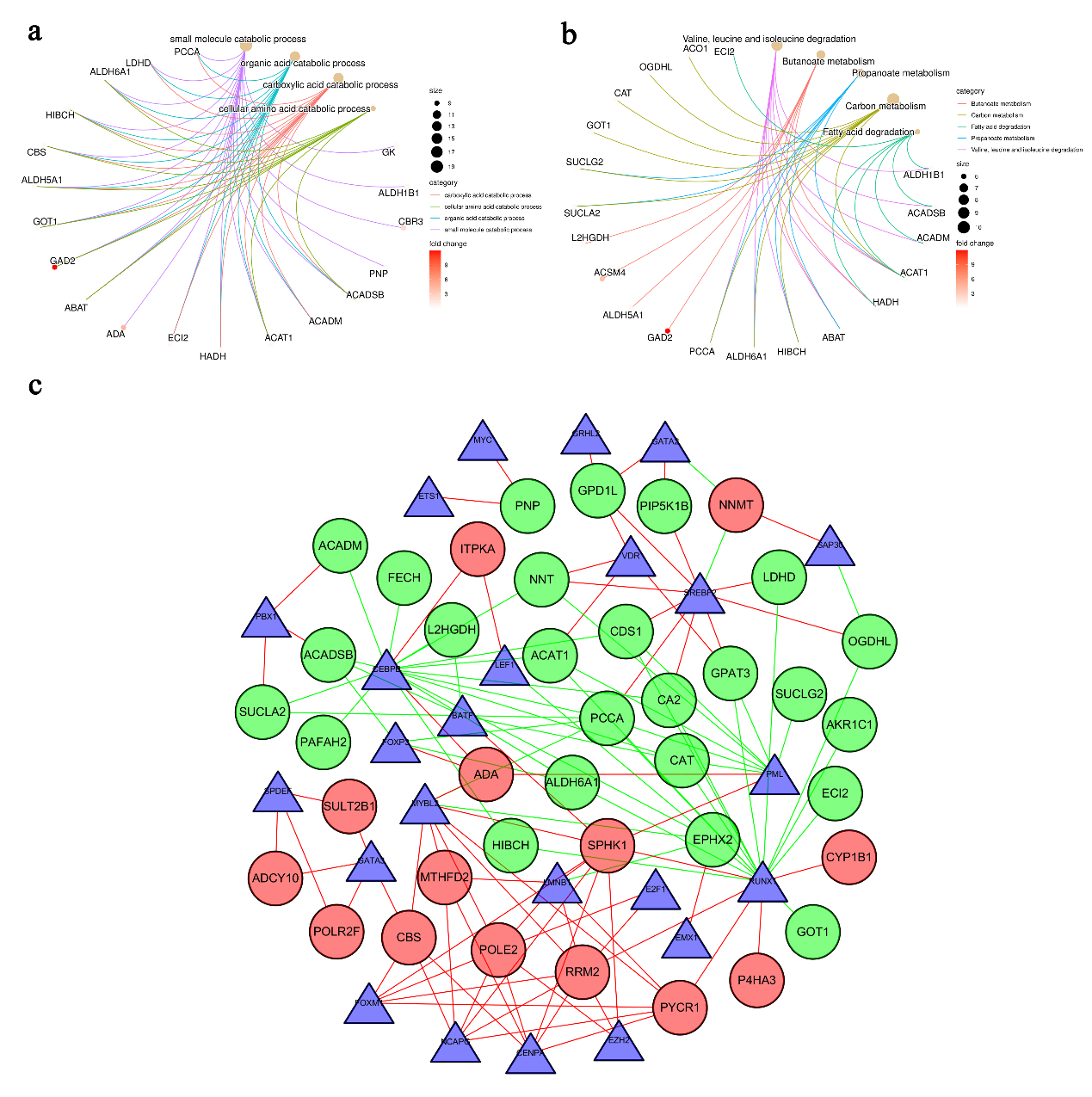


**Fig.S2** LASSO regression analysis to select hub MRGs. (a) Profile of the LASSO coefficient for each prognostic MRG. (b) Plot of partial likelihood deviance.


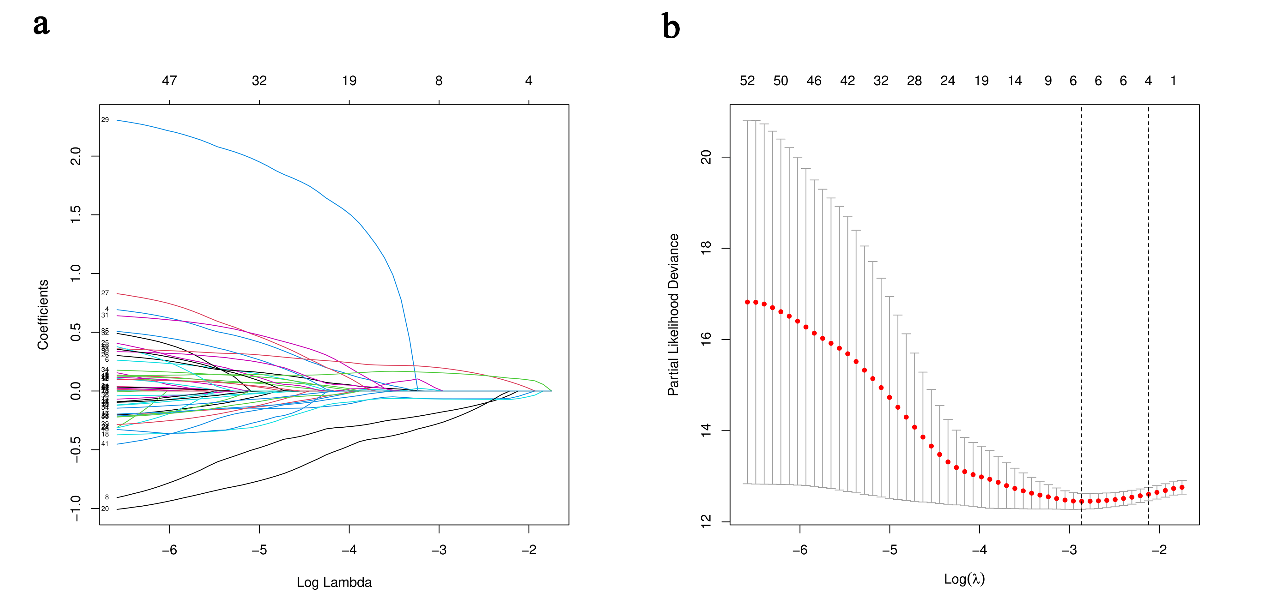


**Fig.S3** Verification of the 6-MRG risk signature with OS of ccRCC. (a-b) MRG risk signature verification of Kaplan-Meier curve and ROC curve in the GSE22541 cohort. (c-d) MRG risk signature verification of Kaplan-Meier curve and ROC curve in the FAHWMU cohort.


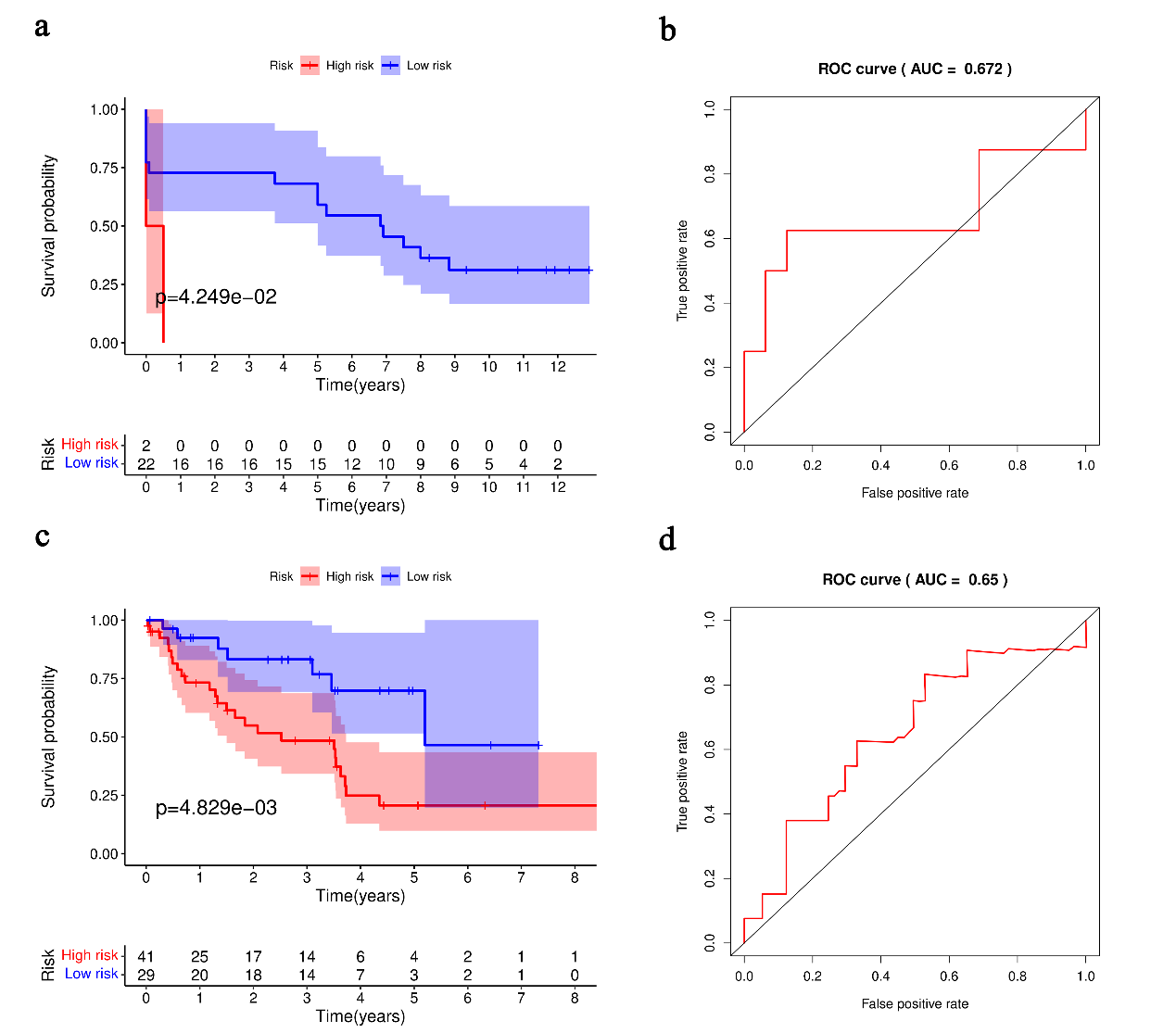


**Fig.S4** The correlation of 6 MRGs in the risk signature with OS in ccRCC. Kaplan-Meier curves of PAFAH2 (a), ACADSB (b), ACADM (c), HADH (d), PYCR1 (e) and ITPKA (f).


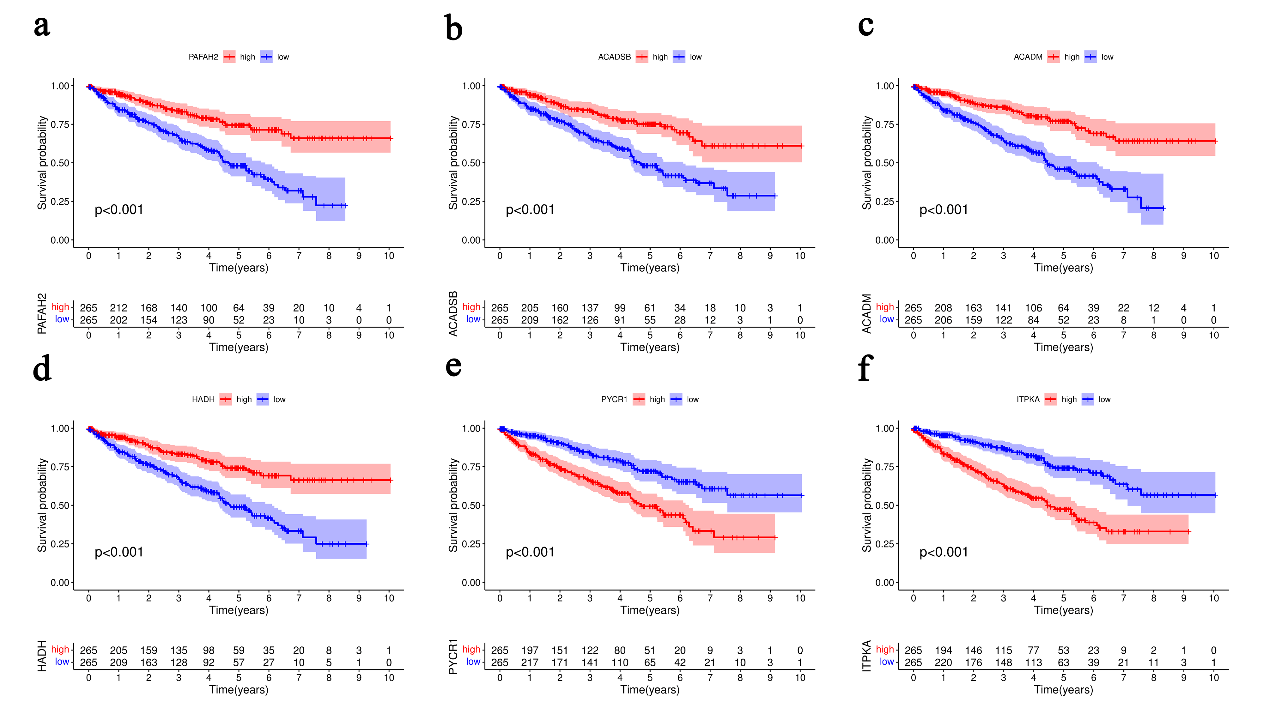


**Fig.S5** Independent prognostic analysis and establishment of nomogram. Univariate (a) and multivariate (b) cox regression analysis between clinical characteristics and risk scores. (c) A nomogram based on age and risk score in ccRCC. (d) The calibration plots of nomogram within 1-, 2- and 3-year, respectively.


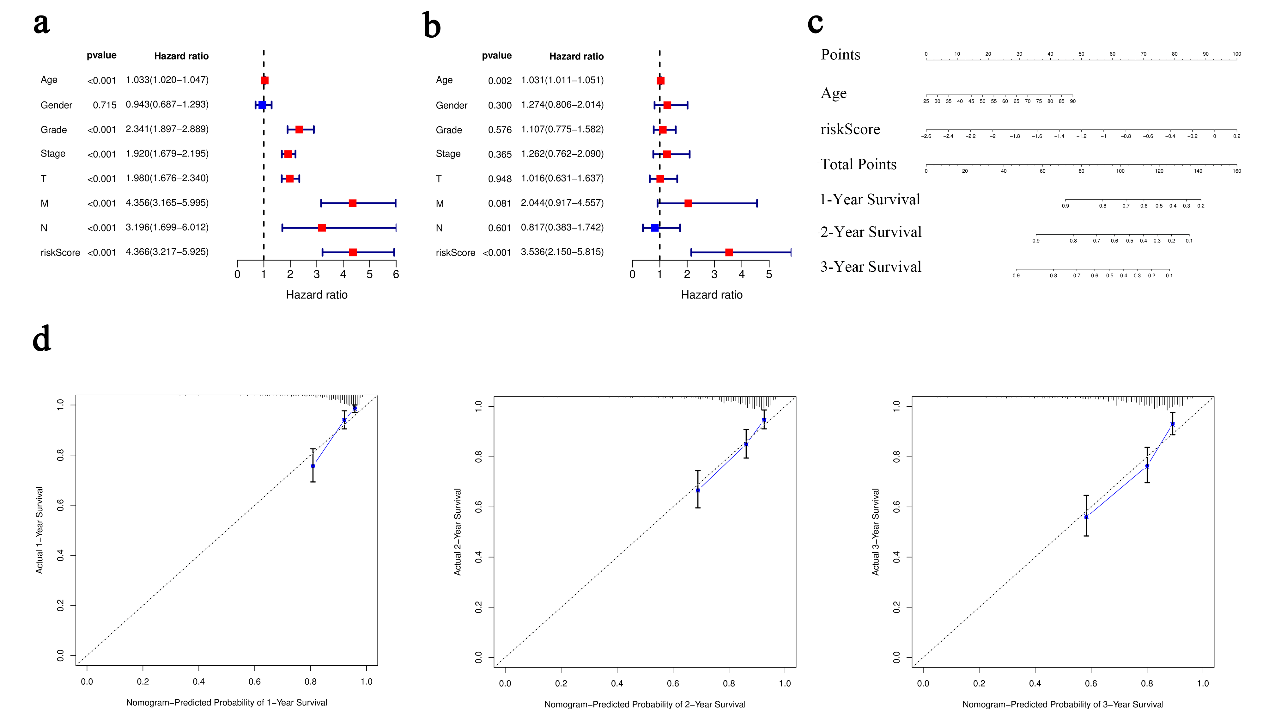


**Fig.S6** The protein levels of 5 MRGs in the risk signature in the database of Clinical Proteomic Tumor Analysis Consortium. The boxplots of PAFAH2 (a), ACADSB (b), ACADM (c), HADH (d) and PYCR1 (e) between adjacent normal kidney tissue and tumor tissue.


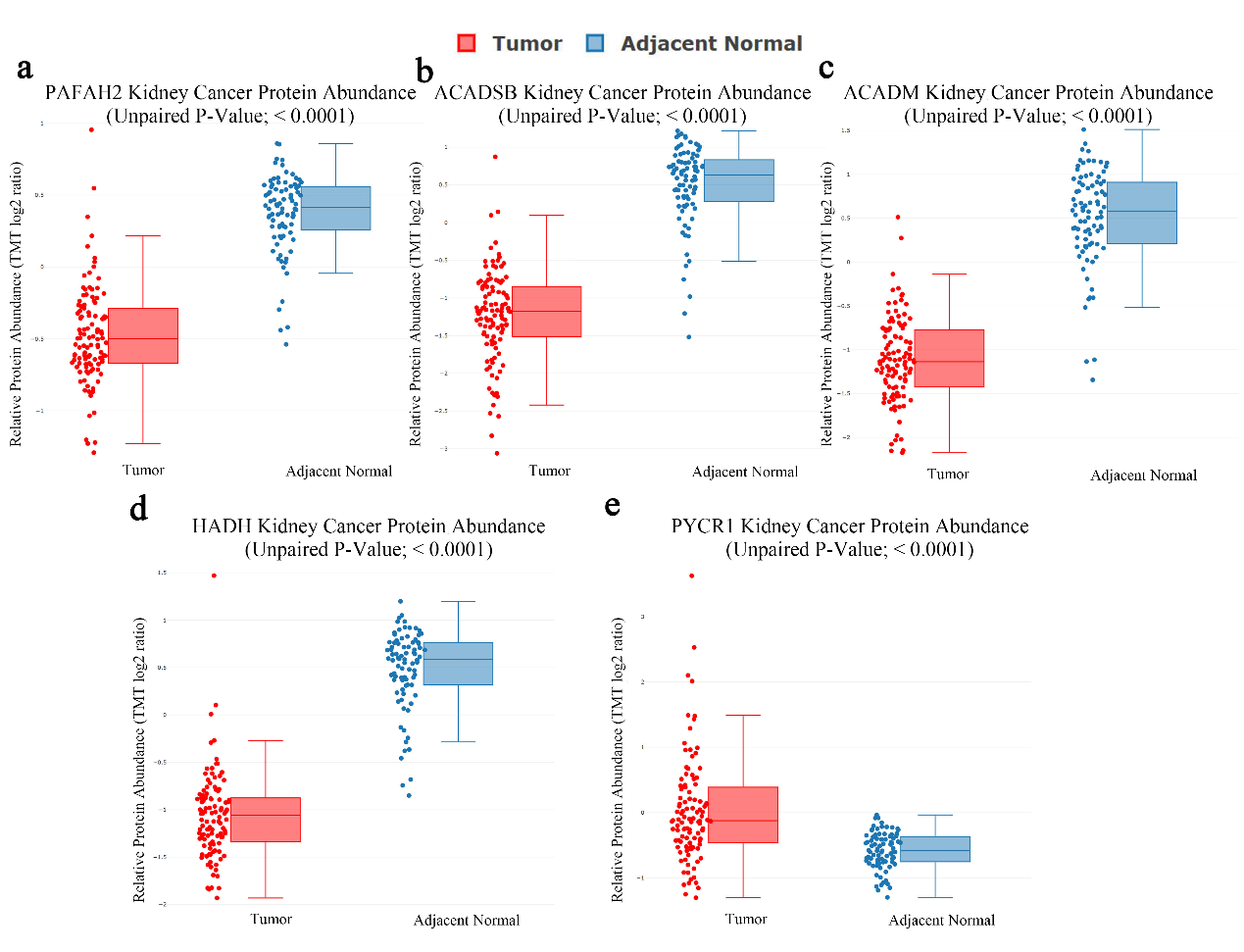

Supplement: Supplementary file 1 — Supplementary Information. [file 41598_2023_38380_MOESM1_ESM.docx]
